# Efficient Characterization of Parametric Uncertainty of Complex (Bio)chemical Networks

**DOI:** 10.1371/journal.pcbi.1004457

**Published:** 2015-08-28

**Authors:** Claudia Schillings, Mikael Sunnåker, Jörg Stelling, Christoph Schwab

**Affiliations:** 1 Seminar for Applied Mathematics, ETH Zürich, Zürich, Switzerland; 2 Department of Biosystems Science and Engineering and SIB Swiss Institute of Bioinformatics, ETH Zürich, Zürich, Switzerland; The Pennsylvania State University, UNITED STATES

## Abstract

Parametric uncertainty is a particularly challenging and relevant aspect of systems analysis in domains such as systems biology where, both for inference and for assessing prediction uncertainties, it is essential to characterize the system behavior globally in the parameter space. However, current methods based on local approximations or on Monte-Carlo sampling cope only insufficiently with high-dimensional parameter spaces associated with complex network models. Here, we propose an alternative deterministic methodology that relies on sparse polynomial approximations. We propose a deterministic computational interpolation scheme which identifies most significant expansion coefficients adaptively. We present its performance in kinetic model equations from computational systems biology with several hundred parameters and state variables, leading to numerical approximations of the parametric solution on the entire parameter space. The scheme is based on adaptive Smolyak interpolation of the parametric solution at judiciously and adaptively chosen points in parameter space. As Monte-Carlo sampling, it is “non-intrusive” and well-suited for massively parallel implementation, but affords higher convergence rates. This opens up new avenues for large-scale dynamic network analysis by enabling scaling for many applications, including parameter estimation, uncertainty quantification, and systems design.

## Author Summary

In various scientific domains, in particular in systems biology, dynamic mathematical models of increasing complexity are being developed and analyzed to study biochemical reaction networks. A major challenge in dealing with such models is the uncertainty in parameters such as kinetic constants; how to efficiently and precisely quantify the effects of parametric uncertainties on systems behavior remains a question. Addressing this computational challenge for large systems, with good scaling up to hundreds of species and kinetic parameters, is important for many forward (e.g., uncertainty quantification) and inverse (e.g., system identification) problems. Here, we propose a sparse, deterministic adaptive interpolation method tailored to high-dimensional parametric problems that allows for fast, deterministic computational analysis of large biochemical reaction networks. The method is based on adaptive Smolyak interpolation of the parametric solution at judiciously chosen points in high-dimensional parameter space, combined with adaptive time-stepping for the actual numerical simulation of the network dynamics. It is “non-intrusive” and well-suited both for massively parallel implementation and for use in standard (systems biology) toolboxes.

This is a *PLOS Computational Biology* Methods paper

## Introduction

Chemical reaction networks (CRNs) form the basis for analyzing, for instance, cell signaling processes because they capture how molecular species such as proteins interact through reactions, for example, to form larger macromolecular complexes. In the limit of (sufficiently) high copy numbers of the molecular species when stochasticity can be ignored [1], the dynamic behavior of a CRN is described by a *parametric*, nonlinear deterministic system of ODEs of the form (see, e.g., [2] and references therein):

$$\frac{dx\left(t\right)}{dt}=f\left(x\left(t\right),u\left(t\right),p\right)=N\;v\left(x\left(t\right),u\left(t\right),p\right),x\left(t_{0}\right)=x_{0},$$ (1)

where $x(t)\;\in 𝓢=IR_{\geq 0}^{n_{x}}$ is the vector of the non-negative concentrations of the *n*_*x*_ molecular species that depend on time *t*, *f*(*x*(*t*), *u*(*t*),**p**) is a system of *n*_*x*_ functions that model the rate of change of the species concentrations depending on the current system state *x*(*t*) and on the parameter vector $\text{p }={\left(p_{k}\right)}_{k=1}^{n_{p}}\in \;IR_{\geq 0}^{n_{p}}$ of dimension *n*_*p*_ which equals the number of kinetic parameters (physical constants) associated with the biochemical reactions. The inputs $u(t)\;\in IR^{n_{u}}$ may be time-varying, for example, when external stimuli to signaling networks are being considered. The initial conditions are given by *x*_0_. Here, we follow the notational conventions of the application domain; the mathematical literature usually denotes states and parameters by *x* and *y*, respectively. For CRNs, specifically, the right-hand-side *f*(*x*(*t*), *u*(*t*),**p**) can be decomposed into two contributions: the stoichiometric matrix $N\in IR^{n_{x}\times n_{r}}$ that encodes how species participate in reactions (its entries correspond to the relative number of molecules of each of the *n*_*x*_ species being consumed or produced by each of the *n*_*r*_ reactions), and the vector of *n*_*r*_ reaction rates, or fluxes, $v(x(t),u(t),\text{p})\;\in IR_{\geq 0}^{n_{r}}$.

Using ODE models Eq (1) to analyze cellular networks is challenging, in particular, because *n*_*p*_ is large and the parameter values are usually unknown. For instance, enzyme kinetic parameter values are distributed over several orders of magnitude [3], making it often difficult to ascertain even rough estimates when the parameter values cannot be determined experimentally. In practice, parameter values need to be estimated from experimental observations such as time-course data of species concentrations, which typically involves solving computationally expensive global optimization problems [4]. In addition, mainly due to limited measurement capabilities and a still prevailing shortage of quantitative experimental data, most of the established (systems biology) models have ‘sloppy’ parameters. That is, their values are not sufficiently constrained by the data used for estimation, or some parameters are even redundant, for a given set of measurement data. These parametric uncertainties may propagate to large uncertainties in model predictions [5, 6]. In parameter estimation and uncertainty quantification, one needs to determine how the system behavior *x*(*t*) depends on the parameters **p**, ideally on the entire (physically feasible) parameter space. While local evaluations in parameter space may suffice in certain cases, for instance, methods for Bayesian inference of model parameters and topologies [7, 8] are global by design, making the last aspect a critical requirement.

**Fig 1:**
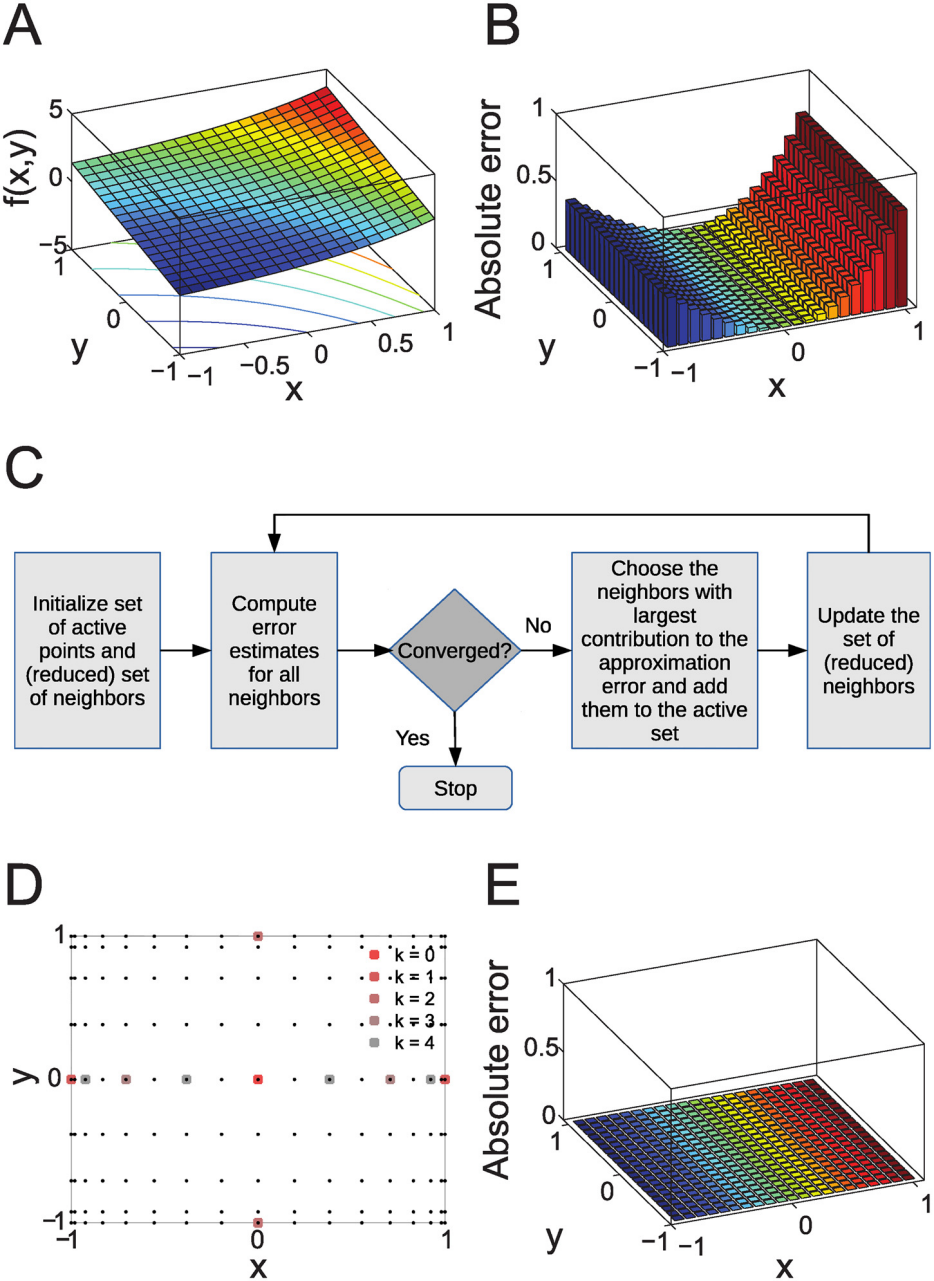
Example model. **A:** The function *f*(*x*, *y*) = *exp*(*x*) + *y* plotted in the region −1 ≤ *x*, *y* ≤ 1. **B:** Absolute difference between the original function (**A**) and the first order approximation $f(x,y)\approx f(0,0)+{x\frac{\partial f(x,y)}{\partial x}|}_{\left(0,0\right)}+{y\frac{\partial f(x,y)}{\partial y}|}_{\left(0,0\right)}=1+x+y$. **C:** Outline of the algorithm for the proposed adaptive sparse interpolation method. **D:** The 11 interpolation points computed by the Smolyak algorithm for five (*k* = 0…4) iterations for an error tolerance of 5 × 10^−8^. Possible Smolyak grid points up to the same level of hierarchy are shown as black dots. **E:** Absolute difference between the original function (**A**) and the Smolyak approximation based on the interpolation points in (**D**), computed for 441 uniformly distributed points in −1 ≤ *x*, *y* ≤ 1.

In systems biology (most of the ensuing considerations apply beyond systems biology), two broad classes of approaches to computational quantification of parametric uncertainty can be distinguished. So-called *local methods* rely on parameter sensitivities

$$s_{k}(t,\text{p})={\frac{\partial x(t)}{\partial p_{k}}|}_{\text{p}={\text{p}}_{0}}$$ (2)

that provide first-order approximations of the systems’ behavior when the *k*-th parameter, *p*_*k*_, has small variations around the nominal parameter set ${\text{p}}_{0}\;=\;{\left(p_{0k}\right)}_{k=1}^{n_{p}}$. Parameter sensitivities allow for an assessment of, for instance, metabolic network behavior in response to small parametric perturbations [9]. However, as systems biology models are typically highly non-linear, and calibrations to noisy data may access parameter values that are far from **p**_0_, the scope of local approximations is limited. For example, the response of the two-dimensional example model shown in Fig 1A appears ‘simple’, but a first-order approximation of the response becomes increasingly inaccurate with increasing distance from the nominal parameter set (Fig 1B).

*Sampling-based methods*, in contrast, attempt to cover the entire parameter space. For large networks, high-dimensional parameter spaces need to be explored, and due to the so-called “curse of dimensionality” [10], this entails sample numbers (and thus, computation time) that increase exponentially with the dimension of the parameter space. In addition, limited prior knowledge on parameter regimes and location of disconnected regions in parameter space often limit targeted or adaptive sampling strategies. State of the art Monte-Carlo methods have been reported to cope with up to 50 model parameters [8, 11], but present CRN models in systems biology may have several hundred parameters [12]. Hence, not only for the efficient computational forward and Bayesian inversion analysis of large-scale models representing entire cells [13], but also for pathway models [14], efficient computational methods with mathematically founded, favorable scaling of work versus accuracy with respect to the model size are lacking.

One possible avenue for developing more efficient computational methods consists of exploiting specific features of the application domain (models), which proved successful for determining local parameter sensitivities [15]. For CRN models which arise in systems biology such as Eq (1), one can exploit that many cellular reaction networks are only weakly connected. This is reflected in *sparse* (but not block diagonalizable) stoichiometric matrices *N*, and in the scale-free structure of many large-scale networks that comprise a few hubs with many connections, whereas most species have few connections [16]. In addition, if one considers only mass-action kinetics, the reaction rates can be written as

v(x(t),u(t),p)=diag(p)ρ(x(t))+Ou(t),

where $O\in {\mathbb{N}}^{n_{r}\times n_{u}}$ defines the input-to-rate mapping and *ρ*(*x*(*t*)) is a vector of monomials in the states *x*(*t*) [17], revealing an overall *affine parameter dependence* of *f*(*x*(*t*), *u*(*t*),**p**).

Here, we propose a novel, adaptive deterministic computational methodology for handling parametric uncertainty for high dimensional parameter spaces with particular attention to large, parametric nonlinear dynamical systems in CRN models. We exploit recent mathematical results [18] stating that responses of systems models with *sparse* and *affine* dependence on these parameters can be captured by sequences of polynomial approximations such that the approximated responses converge to the exact responses with rates that are independent of the dimensions of the parameter and state space. The presently proposed approach adaptively exploits this sparsity. It provably allows to adaptively scan system responses across the entire, high-dimensional parameter space with less instances of (possibly costly) forward simulations than with sampling methods to reach prescribed numerical accuracies of the responses. It also allows to build parsimonious parametric surrogate models that are valid over the entire parameter space. To demonstrate our methodology’s performance, we apply it to three published systems biology models, where the numerical results support the theoretical prediction of dimension-independent convergence rates beyond the rate 1/2 for Monte-Carlo sampling methods.

## Methods

### Overview

We propose an adaptive deterministic algorithm that relies on constructing sparse interpolation and quadrature grids in high-dimensional parameter spaces as outlined in Fig 1C. It relies on so-called Smolyak sparse grids [19] that exploit that for functions in high dimensions, not all parameter points are equally important to approximate the function. The Smolyak method can employ different sequences of univariate quadrature formulae; here, we focus on the generation of grid points using the Clenshaw-Curtis method (CC; see S1 Text for details). Correspondingly, the principle of our adaptive Smolyak sparse grids method is to start from a single parameter point and to iteratively evaluate the effect of adding neighboring points in certain directions of the parameter space, until we fall below a predefined numerical error tolerance. Note that here and in the following, ‘error tolerance’ refers to numerical accuracy and not to model properties such as robustness. This principle is illustrated in Fig 1D for the two-dimensional example model, where *k* denotes the iteration. In particular, the directions in which the most points are added correspond to the parameters for which the model is the most responsive. Once the points to be added (‘activated’) are determined for one iteration, simulations to determine the function values are independent of each other, allowing for a parallelization of computations. Note, that the effect of adding points in more than one parameter space direction simultaneously is not evaluated, since this is (often) computationally intractable. However, for certain functions such as the example model, the approximation resulting from few (five, in this case) iterations may be highly accurate over the entire domain in parameter space (Fig 1E). In the following, we focus on why subsets of CRN models allow for sparse interpolation and quadrature with dimension-independent convergence (numerical error tolerance) properties, and for mathematical details we refer the reader to the S1 Text and to [18, 20]. Note also that an implementation of the method (for model 1 discussed in the Results section) is available as S1 File.

### Models with mass-action kinetics

We consider models of the form of Eq (1) with reactions based on mass-action kinetics. For physically realistic reactions with at most two educts and a bounded parameter domain, this implies: $v_{j}(x,u,\text{p})=p_{j}\rho_{j}(x)+\sum_{k=1}^{n_{u}}o_{jk}u_{k}=p_{j}x_{l}x_{m}+\sum_{k=1}^{n_{u}}o_{jk}u_{k}$, *j* = 1, …, *n*_*r*_, for some given indices (depending on j) *l*, *m* ∈ [1, …, *n*_*x*_], where the parameters *p*_*j*_ ≥ 0 and *p*_*j*_ ∈ [*a*_*j*_, *b*_*j*_]. To save space we write *x* ≔ *x*(*t*) and *u* ≔ *u*(*t*). The right-hand-side of the ODE for state variable *x*_*i*_, *i* ∈ [1, …, *n*_*x*_], is:

$$f_{i}\left(p,x,u\right)=\sum_{j\geq 1}n_{ij}v_{j}=\sum_{j\geq 1}n_{ij}p_{j}\rho_{j}\left(x\right)+\sum_{j\geq 1}n_{ij}\sum_{k=1}^{n_{u}}o_{jk}u_{k},$$ (3)

where *n*_*ij*_ and *o*_*ij*_ are the elements on row *i* and column *j* of *N* and *O*, respectively. For models of the form of Eq (3), the solution *x*(*t*,**p**) may be approximated with a *surrogate model* based on truncated polynomial expansions in parameter space.

### Parameter scaling

The adaptive sparse quadrature approach requires parameter ranges that are of unit size, and symmetric about zero. To this end, we rescale the parameters by an affine reparametrization: $p_{j}=\frac{b_{j}-a_{j}}{2}{\tilde{p}}_{j}+\frac{b_{j}+a_{j}}{2}$, where ${\tilde{p}}_{j}\in [-1,1]$. Then, with $\phi_{:j}(x)=n_{:j}\frac{\left(b_{j}-a_{j}\right)}{2}\rho_{j}(x)$, denoting by *n*_:*j*_ the jth column of *N*, Eq (3) takes the form:

$$\begin{array}{cc} f_{i}\left(\cdot \right)= & \underset{j\geq 1}{\sum }{\tilde{p}}_{j}\phi_{ij}\left(x\right)+\underset{=:\phi_{i0\left(x,u\right)}}{\underset{︸}{\underset{j\geq 1}{\sum }n_{ij}\frac{b_{j}+a_{j}}{2}\rho_{j}+\underset{j\geq 1}{\sum }n_{ij}\overset{n_{u}}{\underset{k=1}{\sum }}o_{jk}u_{k}}} \end{array}$$ (4)

where the last two terms summarized by *ϕ*_*i*0_(*x*, *u*) are independent of the model parameters. The domain of the parameters is then given by the Cartesian product $U\;=\;{\left[-1,\;1\right]}^{n_{p}}$.

### Adaptive Smolyak sparse grids

Assume an infinite number of terms in Eq (4). Now let *σ* be the maximal value of *s* for which $\sum_{j=1}^{\infty }|L_{j}{|}^{s}<\infty$ holds, where *L*_*j*_ is the Lipschitz constant of *ϕ*_*j*_ (i.e., $\frac{\left\|\phi_{j}(x)-\phi_{j}(x^{\prime })\right\|}{\left\|x-x^{\prime }\right\|}\leq L_{j}$ for ∀*x* ∈ *U*(*x*′), where *U*(*x*_0_) is the neighborhood of any feasible state vector *x*_0_). The approximation error (difference between the original model and the computational surrogate model) is then bounded by *CM* ^−*r*^, where *M* denotes the number of forward simulations, $r=\frac{1}{\sigma }-1$ and 0 < *σ* < 1 and *C* > 0 is a constant that is independent of the system size [18]. Furthermore, the Lipschitz constants for *ϕ*_*j*_(*x*) can be made arbitrarily small by adjusting the distance between *a*_*j*_ and *b*_*j*_ due to the rescaling of the parameter range. The performance of the adaptive Smolyak method typically improves once we constrain admissible parameter ranges to small neighborhoods near nominal values.

For CRN models the number of reaction terms in Eq (3) is finite, but possibly (very) large. Then the error bound *CM* ^−*r*^ obtained in [18] in the infinite-dimensional case is valid, with *C* and *r* independent of the system size. Importantly, the *convergence rate* *r* is independent of the dimension of the parameter space (the number of model parameters). It depends only on the sparsity *σ* ∈ (0,1) afforded by a system’s kinetic description. Here, the term *sparsity* does *not* refer to sparsity in the CRN connectivity graph, but to the frequency of appearance of large coefficients in (generalized) polynomial chaos expansions (‘gpc’ expansions, for short) of the parametric systems’ responses; it is mathematically encapsulated as “*p*-summability of the gpc coefficient sequence”. This has recently been established for high-dimensional CRN models based on mass-action kinetics [18]. There, a large number of “almost” decoupled subsystems increases sparsity in polynomial expansions of parametrized system responses, which is favorable for performance of our adaptive Smolyak algorithms. This convergence rate should be compared to that of conventional tensor product interpolation methods, which decreases with the dimension *n*_*p*_ of the parameter space. For illustration, consider the following linear model (see [20] for numerical experiments):

$$\frac{dx}{dt}=\sum_{j=1}^{\infty }p_{j}j^{-s}x+u,$$ (5)

where *s* > 1, and the number of parameters is infinite. By comparing Eq (5) to Eq (3) we have that: *ϕ*_*j*_(*x*) = *j* ^−*s*^ *x*. Therefore the Lipschitz constant *L*_*j*_ for *ϕ*_*j*_(*x*) is *j* ^−*s*^, and:

$$\sum_{j=1}^{\infty }{\left|L_{j}\right|}^{\sigma }=\sum_{j=1}^{\infty }{\left(j^{-s}\right)}^{\sigma }=\sum_{j=1}^{\infty }j^{-s\sigma }.$$ (6)

It is well known that the series $\sum_{j=1}^{\infty }j^{-q}$ converges for *q* > 1 [21]. Therefore the sum in Eq (6) converges for *sσ* > 1 and for $\sigma >\frac{1}{s}$. Note that the larger the value of *s*, the smaller the potential values of *σ*, and the larger the convergence rate: $r=\frac{1}{\sigma }-1$.

### Surrogate models

With the final surrogate model, we can compute the expected value (and possibly higher moments) for modeled system properties. Typically, system properties that have not been (or cannot be) experimentally measured are of interest. The expected value of a quantity Φ(**p**), in the rescaled parameter region *U*, reads:

$$E\left[\Phi \left(p\right)\right]=\int_{U}\Phi \left(p\right)p\left(p|D\right)dp=\int_{U}\Phi \left(p\right)\frac{p(D|p)p(p)}{p(D)}dp$$ (7)

where *D* are the experimental data, *p*(**p**∣*D*) is the posterior distribution given data *D*, *p*(*D*∣**p**) is the likelihood, and *p*(**p**) is the prior distribution. We assume additive, Gaussian observation noise. The measurement model for *K* experimental observables and *n*_*t*_ time instances is of the form: *y* = *h*(**p**) + *η*, *η* ∼ 𝓝(0,Γ). The likelihood then takes the form of a (inverse) covariance-scaled least squares functional $p(D|\text{p})∼\;\prod_{k=1}^{n_{t}}\text{exp}(-\frac{1}{2}{\left(y_{k}-h_{k}(\text{p})\right)}^{T}\Gamma_{k}^{-1}(y_{k}-h_{k}(\text{p}))$, where *y*_*k*_ ∈ *D* is the data at observation time *t*_*k*_. Marginalizing over the parameter space, we compute the evidence *p*(*D*) as

$$p\left(D\right)=\int_{U}p\left(D,p\right)dp=\int_{U}p\left(D|p\right)p\left(p\right)dp.$$ (8)

Such an explicit computation of the evidence is computationally inexpensive for surrogate models based on sparse gpc approximations (it may not be necessary for all applications, however). Sparsity in the parametric solution of Eq (1), with the right-hand-side defined in Eq (3), implies sparsity in the parametric posterior distribution. Hence, the integral in Eq (8) (and Eq (7)) computed with an output-adapted sparse grid with *M* points converges with rate *CM* ^−*r*^ where *C* > 0 and *r* depends only on the sparsity *σ*, as discussed above. This should be compared to the Monte Carlo approach (e.g. [22]). Here, the expected value of Φ(**p**) is estimated by the finite sample average

$$E_{M}\left[\Phi \left(p\right)\right]≔\frac{1}{M}\sum_{i=1}^{M}\Phi \left(p_{i}\right)$$ (9)

where the sequence of parameter samples **p**_*i*_, *i* = 1, …, *M* is i.i.d drawn from the posterior distribution *p*(**p**∣*D*) (e.g., see the randomized Metropolis-Hastings Markov chain Monte Carlo (MH-MCMC) method [23]). The asymptotic convergence rate of the sample average Eq (9) as the number *M* of samples (i.e., the number of forward simulations) tends to ∞ is bounded by

$$∥E_{M}{\left[\Phi \left(p\right)\right]-E\left[\Phi \left(p\right)\right]∥}_{L^{2}}\leq M^{-1/2}{∥\Phi \left(p\right)∥}_{L^{2}}.$$ (10)

The (mean square w.r.t. the prior) convergence rate 1/2 (to be distinguished from the actual computational work, which increases linearly with the number of parameters) Eq (10) is also independent of the dimension of the parameter space. However, this rate is low (at most = 0.5, implying in particular that error reduction by a factor 1/2 mandates four times as much work) compared to the convergence rate afforded by the adaptive Smolyak process.

## Results

To validate the implementation of the dimension-adaptive Smolyak algorithm and to quantify its performance for CRN models, we applied it to three published systems biology models that range from small-scale to one of the highest-dimensional current models using *in silico* generated data.

### Model 1: Glucose uptake in yeast

The availability of nutrients plays a major role for the survival, growth, and proliferation of microorganisms such as the yeast *Saccharomyces cerevisiae*. Glucose specifically is imported into the cells and directly processed in the glycolytic pathway. Yeast prefers glucose over other carbon sources such as fructose and mannose and it therefore possesses intricate mechanisms for glucose sensing. However, the initial mechanisms for glucose sensing and activation have often turned out to be more difficult to elucidate than downstream components and their functions [24].

**Fig 2:**
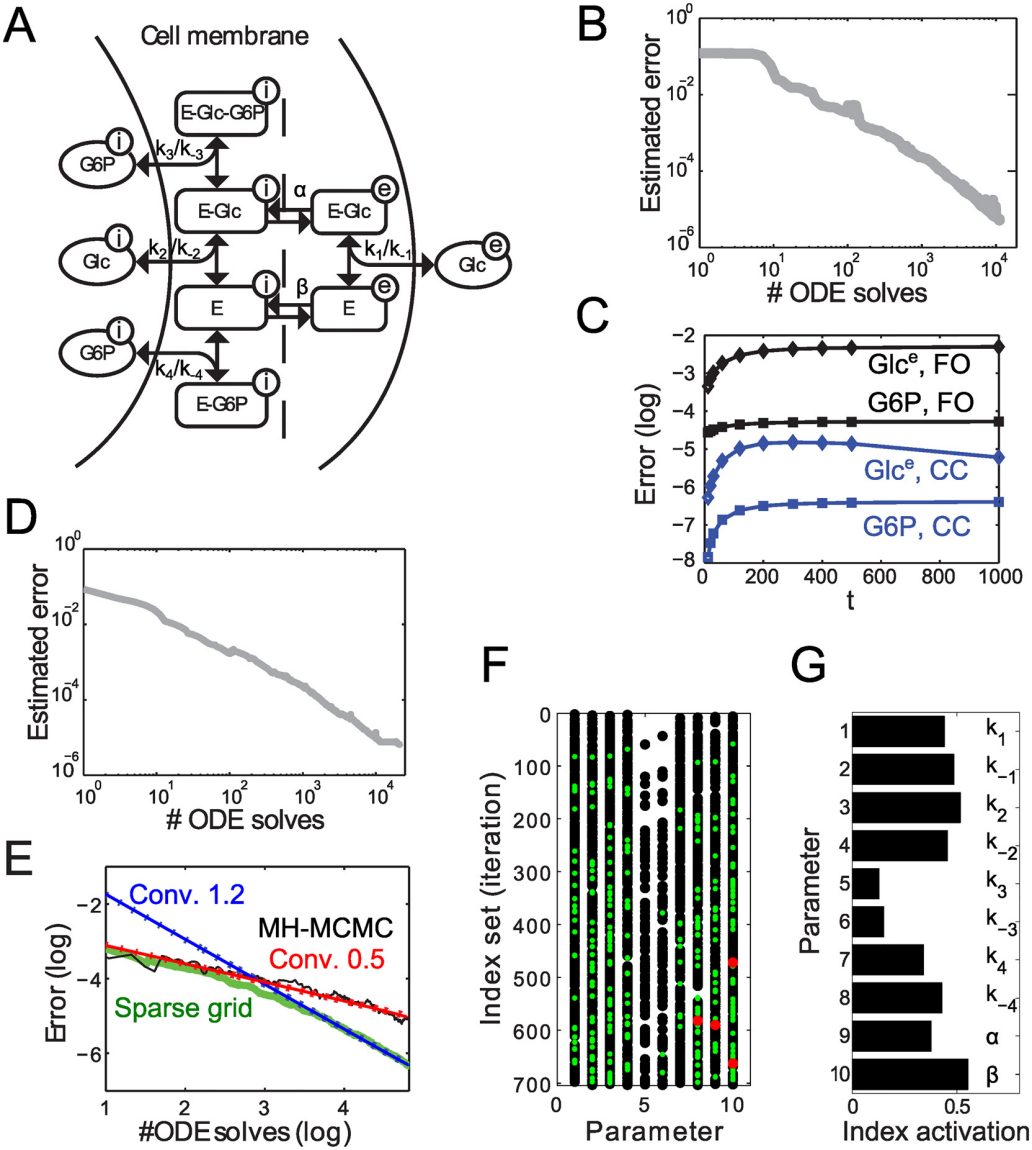
Analysis of the glucose model. **A:** Model for glucose uptake in *S. cerevisiae* cells. Protein E transports glucose (Glc) between the external (e) and internal (i) regions of the cell membrane. Glucose-6-phosphate (G6P) inhibits the uptake of intracellular glucose at the membrane. Model parameters such as association (*k*_*i*_) and dissociation (*k*_−*i*_) constants are indicated next to the corresponding reaction arrows. **B:** Estimated maximal (absolute) errors in the interpolation (Clenshaw-Curtis, CC) over three state variables (external and internal glucose, and G6P), and over time, w.r.t. the number of ODE solves in the parameter region ±0.25**p**_0_ (normal space). **C:** Absolute error over time for the first order (FO) approximation and the sparse grid (CC) solution, from comparisons to the exact ODE solution at a randomly chosen parameter point in ±0.25**p**_0_ (normal space). The result is representative in the investigated parameter region (cf. S1 Text). Approximation errors (log_10_) for external glucose are represented by blue (CC) and black (FO) diamonds, and for G6P by blue (CC) and black (FO) squares. **D:** Estimated maximal (absolute) errors in the normalization constant for Bayesian inference for the same settings as in (**B**). **E:** Comparison of convergence rates for the adaptive Smolyak approach (green) and MH-MCMC (black). For MH-MCMC the normalized error was computed as the maximal (absolute) error between approximation and exact solution (approximated by 100.000 samples), at the time points in (**C**), for the six state variables involving transporter E (**A**). The red (blue) line indicates a convergence rate of 0.5 (1.2). **F:** Computed index sets for ≈ 700 iterations of the adaptive Smolyak algorithm for interpolation (CC, ±0.25 ⋅ **p**_0_), w.r.t. the 10 model parameters. Each dot represents an increased number of grid points in the direction of the corresponding parameter and the color indicates the order of the interpolation formula: black = 1; green = 2; red = 3. **G:** Activation of indices (that is, number of grid points) per parameter direction, normalized by the number of iterations. Parameter identifiers correspond to (**F**) and (**A**), respectively.

A predictive model of glycolysis would therefore be of great interest and efforts have already been made in this direction [25]. However, although the stoichiometric properties of glycolysis are well characterized, the kinetics of individual reactions are difficult to infer. A model for the first steps of glycolysis, characterized by facilitated diffusion of glucose into *S. cerevisiae* cells, has been presented in [26]. In a detailed version of this model with 9 states and 10 parameters, which serves as our small-scale test case, glucose import is inhibited by glucose-6-phosphate (G6P) (see Fig 2A, S1 Text for details, and S1 File for an implementation of the adaptive Smolyak method for this model).

In the *forward analysis*, we focused on the effects of changes in parameters on the dynamics of metabolite concentrations (internal and external glucose, internal G6P) that can be measured with state of the art experimental methods such as mass spectrometry [27]. The adaptive Smolyak interpolation of the corresponding model states shows a convergence rate of 1 with respect to the number of ODE solves needed (Fig 2B; see also S1 Text) to achieve the given accuracy (2 × 10^−5^) in terms of the difference between the original and surrogate model uniformly over the parameter space. To investigate how the accuracy of our algorithm compares to a first-order approximation, we conducted a local sensitivity analysis and observed a gain in accuracy of two orders of magnitude, at comparable work (Fig 2C and S1 Text).

Importantly, with the Smolyak method it is also possible to efficiently compute other system characteristics on the entire parameter domain with a prescribed accuracy. We conducted numerical studies on the *inverse problem* in the context of Bayesian parameter estimation. In the glucose model, such estimation may aim at identifying the concentration of individual carrier complexes over time, which are significantly more difficult to measure with available experimental methods. For Bayesian inference, the adaptive Smolyak algorithm shows a similar convergence behavior as in the forward problem (Fig 2D). However, for some levels of noise in the artificial data we observe a slightly worse convergence rate (approximately 0.65 over 10^5^ ODE solves; Fig 2E), because parameter sets with high posterior probability constitute a small part of the total parameter space. We also compared these results to those obtained from running a Metropolis-Hastings Markov chain Monte Carlo (MH-MCMC) algorithm on the same data, resulting in the same posterior distributions and showing that our implementation is accurate. Notably this was achieved with significantly less computational effort than with MH-MCMC (Fig 2E). These results indicate the potential of the Smolyak algorithm for the efficient forward analysis and Bayesian inversion. However, the difference in performance between the algorithms can be expected to be significantly larger for high-dimensional applications.

Finally, we focused on the biological interpretation of the numerical results with respect to the mechanisms for glucose transport that are most relevant (under our particular choices of observations for the forward problem and the selected domain in parameter space). Fig 2F shows the activation of indices (grid points) per parameter dimension in the forward problem. Visually, it is apparent that different parameters required different numbers of interpolation points and interpolation orders. We quantified this behavior by an index activation, that is, the total order of active interpolants normalized by the number of iterations. While overall the index activation is rather homogeneous (Fig 2G), the approximation of the model behavior depends substantially less on parameters *k*_3_ and *k*_−3_, which relate to the forward and backward directions of the reaction for binding of intracellular G6P to the glucose bound carrier (E-Glc) at the inner region of the cell membrane (see Fig 2A). This reaction is part of a hypothesized inhibition of glucose transport by G6P [26], indicating that the reaction may not exist in reality (under the conditions assumed for the numerical analysis). In contrast to first-order sensitivity analysis, this result is not pertinent to a nominal model parametrization only. More generally, this indicates that the proposed Smolyak sparse grid method can be employed for the detailed analysis of parameter dependencies (and eventual model order reduction) of systems biology models.

### Model 2: Epidermal growth factor receptor (EGFR) signaling

To investigate how our method performs for larger, more typical current systems biology models, we applied it to a model of the EGFR pathway response for the first two minutes upon EGF stimulation [28]. This model was used to explain why EGFR phosphorylation peaks at ≈ 30s and returns to low levels at 1–2 min after stimulation, whereas the phosphorylation of other key proteins increases monotonically. Briefly, the model captures short-term signaling induced by EGF in an ‘upstream’ set of reactions leading from EGFR—EGF binding to active (phosphorylated) EGFR dimers. The interactions of the active receptor with its cytoplasmic target proteins consists of three coupled cycles of reactions involving Grb2, Shc, and PLC*γ*, respectively. Theses cycles feed downstream signaling to targets such as Ras and PI3K [28].

The model has 50 kinetic parameters, whose values were determined based on previous reports and biochemical considerations, leading to a reasonable description of the experimental observations [28]. To identify potential targets for external modification of the pathway behavior (e.g., through drugs), it is interesting to investigate the sensitivity of the pathway response to the kinetic parameters. In [28], the system behavior in response to parametric perturbations was reported to be stable “over a wide range of values”, but in the analysis all rate-constants were simultaneously multiplied by a constant factor (×2), which only leads to a “scaling of the time”. With our method, it is possible to investigate the response to variations in any combination of the parameters. This is a major advantage, since information about the importance of parameters and all the possible response patterns is generated.

**Fig 3:**
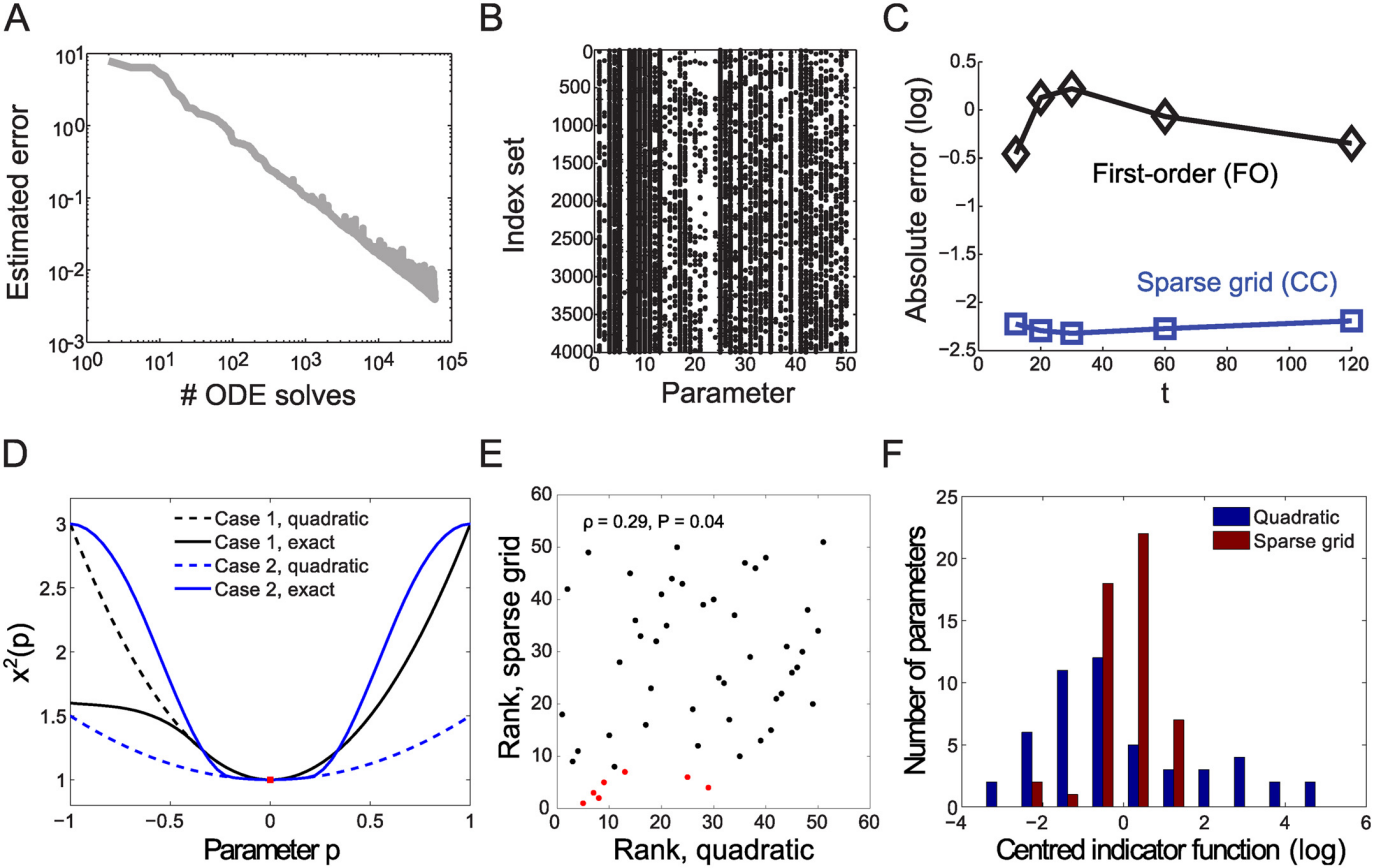
Analysis of the EGFR model. **A:** Estimated maximal absolute error for the adaptive interpolation for states 1–23 with respect to the number of ODE solves (Smolyak, CC, in the region ±0.25 ⋅ **p**_0_, normal space), **B:** Computed index sets for 4000 iterations of the adaptive Smolyak algorithm for interpolation (CC, ±0.25 ⋅ **p**_0_), w.r.t. the 50 model parameters. Each black dot represents an increased number of grid points in the direction of the corresponding parameter. **C:** Maximal absolute errors for the 23 state variables for sparse grids (blue) and FO approximation (black). **D:** Illustration of dependencies of averaged squared changes in model states (*χ* ^2^(*p*)) as a function of a single parameter *p*. A quadratic approximation of the model response to parameter changes around the nominal point (red) can lead to large inaccuracies compared to the exact response when model responses are not symmetric (case 1), or when the local and global behavior are very different (case 2). **E:** Comparison of rank-ordered characterizations of parameter influences by quadratic approximation (eigenvalues of the Hessian matrix) and by the sparse grid method (index activation, as in Fig 2G). Pearson’s correlation *ρ* and corresponding P-value are given at the top; red dots highlight the seven most influential parameters identified by sparse grids. **F:** Sloppiness of model parameters as evaluated by the eigenvalues of the Hessian matrix (quadratic approximation at the nominal point) and by the activation frequency of indices (sparse grid computation). In both cases, distributions were centered by the mean in *log*_10_ space.

The estimated error of the adaptive Smolyak interpolation suggests for this problem a convergence rate of 0.75 with respect to the number *M* of ODE solves needed (Fig 3A). This rather moderate (but still superior to Monte-Carlo sampling) rate results from near isotropic refinement of the sparse interpolant in the 50-dimensional parameter space. This is indicated by the sets of activated indices for the adaptive Smolyak algorithm (Fig 3B), where virtually all parameter dimensions require higher-order approximations. Over extended parameter domains, we again find that our method yields results that are approximately two orders of magnitude more accurate than those obtained by first-order parameter sensitivities (Fig 3C).

The isotropic refinement for sparse grids questions earlier beliefs on generally ‘sloppy’ models in systems biology and in other domains [5, 29] that essentially relied on computing local parameter sensitivities. The analysis of ‘sloppy’ models uses a quadratic approximation of the average squared changes in the model states *χ* ^2^(**p**) at a nominal parameter point. More specifically, the metric for parameter influences proposed are the absolute eigenvalues *λ* of the (quadratic) Hessian matrix; high (low) eigenvalues indicate influential (non-influential) parameters. As illustrated in Fig 3D, however, compared to the exact *χ* ^2^(**p**) that can be computed with our proposed algorithm, the quadratic approximation may be inaccurate when the model response is asymmetric, or when it changes qualitatively distant from the nominal parameter point. The rank-ordered metrics (eigenvalues *λ* for the quadratic approximation and index activation for the sparse grids, respectively) for the EGFR signaling model correlate significantly, but only poorly (Pearson rank correlation *ρ* = 0.29, *P* = 0.04; Fig 3E). The most influential parameters identified by the adaptive Smolyak method, however, yield a biologically consistent interpretation. These parameters pertain to receptor autophosphorylation and dephosphorylation (*k*_3_, *V*_4_, and *K*_4_ in the notation of [28]) as well as active receptor interactions with its direct binding partners Shc (*k*_13_ and *k*_15_) and PLC*γ* (*k*_5_ and *k*_7_). This indicates that control of active receptor by (auto)phosphorylation dominates the model behavior. In contrast, the quadratic approximation would allocate the control to upstream receptor- ligand interactions (*k*_1_, *k*_−1_, *k*_2_, *k*_−2_ are associated with the largest absolute eigenvalues). We find another suggested characteristic of ‘sloppy’ models, namely that eigenvalues spread across many decades [5], also in the EGFR model, but global analysis with a narrowly distributed spectrum of index activations (Fig 3F) again questions the accuracy of local approximations, and interpretations thereof.

Finally, in the Bayesian inverse problem, which consists of computing the conditional expectation of the first state, unbound EGF, under given (artificial) noisy, observational data, the convergence rate was improved to approximately 1 (S1 Text). The improved convergence rate compared to the MH-MCMC method shows the potential of the proposed, adaptive Smolyak approach in particular for larger CRN models with several hundreds of state and parameter variables. We attribute a decrease in the convergence rate for larger parameter variations in the EGFR model (see S1 Text) to the more pronounced impact of nonlinearities in the model. In practical applications such as the Bayesian inference of pathway topologies for EGRF signaling in [8] using models of similar size, however, we expect substantial gains in performance compared to sampling-based methods.

### Model 3: Coupled signaling pathways

To investigate how the adaptive sparse Smolyak method performs in high-dimensional parameter spaces we analyzed a model of the epidermal growth factor (EGF) and heregulin (HRG) activated response in the mammalian ErbB signaling pathways and in the MAPK and Akt cascades [14]. Briefly, the model, formulated entirely in mass-action kinetics, can be seen as a substantial extension of the EGFR model [28] above. It encompasses all four receptor species (ErbB1-4) and their complex interactions explicitly. Degradation pathways via endosomes are represented as well as downstream signaling through the mitogenic Ras/MAPK and the pro-survival PI3K/Akt pathways. Especially the detailed modeling of combinatorial interactions between and at receptor species lead to a model that encompasses 500 states and 229 parameters, making it one of the most complex systems biology models developed to date. In [14], the authors focused again on short-term signaling, and they found that first-order parameter sensitivities are highly context (molecular feature and stimulation condition) specific. However, the model parameters were estimated in a region 2.5 orders below and above the nominal values in log-space. Due to the challenges of parameter identification in high-dimensional, nonlinear ODE models, Chen et al. [14] took a pragmatic approach: model parameters were repeatedly estimated, and patterns in the optimization results were then used to infer model properties in order to cope with the issue of identifying parameters in large parameter spaces, as well as the non-identifiability of the model given the experimental data. However, such an approach does not guarantee that the results represent true model properties—they could be strongly biased.

**Fig 4:**
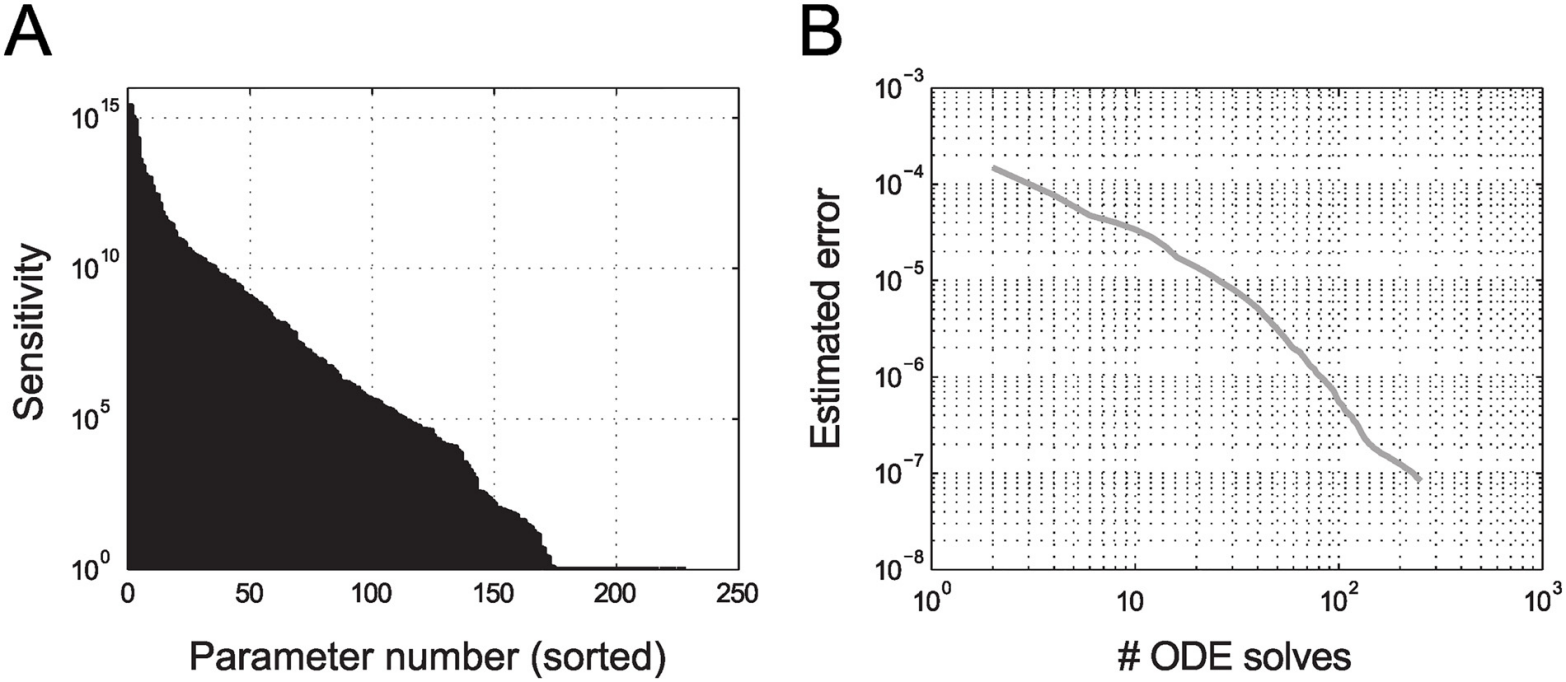
Analysis of coupled signaling pathways. **A:** Sensitivity profile: maximum of the derivative of each state variable w.r.t. each of the parameters ($\frac{dx_{i}}{dp_{j}},i\;=\;1,\;\ldots ,\;n_{x},\;j\;=\;1,\;\ldots ,\;n_{p}$) for all simulated time points and for all 500 state variables. **B:** Estimated maximal absolute error for the adaptive interpolation for all 500 state variables with respect to the number of ODE solves in the region **p**_0_ ± 0.01**p**_0_. Error curves for adaptively expanded regions show a similar trend (see S1 Text).

A detailed sensitivity analysis of this model revealed extreme parameter sensitivities (up to 10^15^), which is summarized in the *sensitivity profile* Fig 4A. The sensitivity profile is an indicator for the sensitivity of the model w.r.t. each parameter, computed as the maximum absolute value of the sensitivity, at the nominal parameter point, over states and time. Such high sensitivity values render a computational forward analysis, as well as Bayesian inference, infeasible even for moderate parameter variations. To cope with such sensitivities, we therefore initially restricted the range of investigated values for each parameter to ±0.01 of the nominal parameter point (**p**_0_). In this region we observe a similar convergence behavior for the adaptive Smolyak interpolation and quadrature as for the two smaller models (Fig 4B). For the computational forward analysis, a gain in accuracy of two orders compared to the first-order approximation and a convergence rate of 1.5 can be achieved (see S1 Text). While we refrain from interpretation of the computational results because of the ill-conditioned model, these performance measures indicate that the proposed adaptive Smolyak method can also make large-scale systems biology models amenable to improved (Bayesian) parameter identification.

We next generated noisy observational data of Akt, Erk, and ErbB phosphorylation at three to four time points for a parameter point in the investigated region. The estimated error of the algorithm indicates a convergence rate of 1–1.5 for the normalization constant of the Bayesian posterior (see S1 Text). In this computation, the adaptive Smolyak algorithm identifies the indices with the largest estimated contribution to the quantity of interest, which can be used in subsequent steps to adaptively enlarge the scanned parameter regions for the less-significant parameters. Hence, we propose the following heuristic strategy for adaptations of the parameter domain: we simply enlarge the parameter variations for all parameters not activated at the current stage of the algorithm. In analyzing model 3, many of the parameters were never activated by the algorithm, indicating that parameter ranges can be made even larger (arbitrarily large for redundant parameters not affecting the response variables). As shown in S1 Text, we obtained promising results with our heuristic strategy, despite the underlying model’s sensitivity issues. We are not aware that sampling-based analysis of a systems biology model of the present scope has ever been achieved.

## Discussion

We propose a sparse, adaptive interpolation scheme for the efficient deterministic computational treatment of parametric uncertainty in complex, nonlinear systems. The methodology is particularly suitable for nonlinear parametric CRN models which commonly appear in computational systems and cell biology. Our numerical analysis of three CRN models that represent the scope of (current) model complexity indicates that the error convergence rate of our method is generically superior to that of Monte Carlo methods, in terms of the number of forward simulations required to reach a prescribed error tolerance. Moreover, MC approaches converge only in mean-square (cf. Eq (10)), whereas the presently proposed methodology delivers “worst-case”, sup-norm convergence rates.

As expected, the efficiency of our method increases when many parameters contribute insignificantly to the model response. When the feasible parameter ranges are narrowed to small neighborhoods of the nominal value due to high sensitivities, our adaptive sparse tensor sampling scheme is superior to the widely used (local) first-order approximations. Also for “well-behaved” models that are equally sensitive to all parameters we observe a higher convergence rate with the Smolyak based approach. In our test problems, the proposed method consistently achieves relative numerical accuracy of five to seven decimals in typical quantities of interest in prediction and Bayesian inference for CRNs. While this accuracy may be considered excessive given the often substantial levels of measurement uncertainty in available data, we assert that *high, certified relative numerical accuracy* is necessary to clearly distinguish computational (e.g., numerical) errors from modeling errors (e.g., erroneous hypotheses on the CRN or on kinetic rate laws), and measurement noise. Our analysis of the EGFR model, for example, demonstrated that numerical parameter dependencies with certified accuracy imply a biological interpretation of sensitive network parts that is different from low-order approximations without such guarantees. Moreover, the postulated prevalence of ‘sloppy’ models in systems biology may need re-evaluation in the light of our findings of nearly isotropic model responses to parameter changes.

Our adaptive Smolyak interpolation method also has several other attractive features. Our method as well as MCMC will exactly characterize parametric dependencies in the limit of infinite samples or grid points. However, unlike MCMC, the sparse interpolation process provides a *reduced surrogate model* upon termination. This model can be quickly evaluated at additional parameter points. Already for moderate-sized models, such as the ERK model, the proposed sparse grid evaluation uses 28 times less CPU time than the ODE solver. Since the surrogate model is based on tensorized polynomial expansions, the computation of distribution moments via (Smolyak) integration of the surrogate model over the parameter space is trivial, thereby overcoming a common computational bottleneck of Bayesian analysis. For example, future work could consider Bayesian parameter estimation for the coupled signaling model to increase the model’s realism.

Further improvements of our Smolyak based method could focus on a systematic approach for increasing the feasible range of parameter values. We took first steps in this direction in the analysis of model 3, where the parameter ranges were iteratively extended, with promising results. Another interesting direction is to construct reduced models in an automated fashion, based on sensitivity analysis and quasi-steady state approximations, in different parts of the parameter space. The glucose model provided one example of this approach, identifying mechanisms that are potentially not relevant for overall glucose transport kinetics. The reduced models could then be analyzed in greater detail, e.g. in larger parameter ranges than the original model.

Our proposed methodology can extend the range of (biochemical) models that are amenable to computational analysis, and thereby the complexity of cellular networks that can be addressed with mathematical models. More generally, recent mathematical results on sparsity in gpc expansions of the parametric system responses for affine-parametric models predict that the proposed methodology can achieve convergence rates larger than 1/2 in terms of the number of forward simulations, free from the so-called “curse of dimensionality” [18]. Sparsity in polynomial chaos expansions of parametric responses due to sparse connectivity patterns in model descriptions appears also in nonlinear models of complex systems in other applications. Our presently proposed methodology for their efficient computational analysis extends, therefore, beyond CRNs from systems biology.

## Supporting Information

S1 Text Supplementary Data. Detailed methods description, mathematical models analyzed, and additional numerical results.
(PDF)

S1 File Supplementary File. Implementation of the adaptive Smolyak sparse grid method for the glucose model (model 1).
(GZ) Click here for additional data file.
