# Supplementary material for: Efficient Characterization of Parametric Uncertainty of Complex (Bio)chemical Networks
Source: PLoS Comput Biol. 2015 Aug 28;11(8):e1004457. doi: 10.1371/journal.pcbi.1004457 (PMC4552555; doi:10.1371/journal.pcbi.1004457)
Supplement: S1 Text — Detailed methods description, mathematical models analyzed, and additional numerical results. (PDF) [file pcbi.1004457.s001.pdf]

S1 Text

Efficient Characterization of Parametric Uncertainty of Complex (Bio)chemical Networks

Claudia Schillings, Mikael Sunnåker,
Jörg Stelling, Christoph Schwab

1 Materials and Methods

For the approximation of the forward and inverse problem based on parametric, nonlinear deterministic systems of ODEs arising in the analysis of cellular networks, we use an adaptive Smolyak-type algorithm described in section 2. The algorithm successively identifies parameters with a large impact on quantities of interest, which leads to an adaptive refinement in the parameter space exploiting the sparsity and nonisotropic behavior of the underlying model. This strategy yields convergence rates independent of the number of unknown parameters and superior to MC based sampling strategies. The nonintrusive nature of the proposed algorithm allows to use available forward solvers and is well-suited for a parallel implementation.

All simulations in this work were performed on the BRUTUS and SC02 computing cluster at ETH Zurich (using up to 128 cores for a parallel simulation).

2 Theory and Proposed Method - Adaptive Smolyak Approach

We briefly review theoretical underpinnings of the proposed numerical method, in particular we make precise the notion of *sparsity* of the parameter dependence of the solution $x(t)$ on the vector $\mathbf{p}$ of parameters. To this end, in this section we make explicit the dependence of $x(t)$ on the parameter sequence $\mathbf{p}$ and write $x(t;\mathbf{p})$. Since we are particularly interested in numerical methods whose performance does not deteriorate for a large number n_p of parameters, we derived in [1] bounds on the approximability of the parametric solution $x(t;\mathbf{p})$ in the case when $n_p = \infty$, i.e. when $\mathbf{p} = (p_j)_{j\geq 1}$ denotes a parameter sequence.

The local Lipschitz conditions for $v(x(t), u(t), \mathbf{p})$ are satisfied, in particular, for $v(x(t), u(t), \mathbf{p}) = \operatorname{diag}(\mathbf{p})\, \rho\,(x(t)) + Ou(t)$ which case occurs in mass-action kinetic models. In this case, the dependence of $v(x(t), u(t), \mathbf{p})$ on $\mathbf{p}$ is affine, i.e. the right hand side is of the form

$$f(x(t), u(t), \mathbf{p}) = \phi_0\,(x(t), u(t)) + \sum_{j\geq 1} p_j \phi_j\,(x(t), u(t)) \tag{1}$$

and the mathematical results of [1] apply. Using that in (1), we may rescale the parameters and the ϕ_j, we assume in the following wlog. that $p_j \in [-1, 1]$ for all $j \in \mathbb{N}$. The results of [1] imply, in particular, that under a smallness condition on the ϕ_j (required to ensure convergence of the series in (1)), there exists a unique solution $x(t;\mathbf{p})$ ([1, Theorem 4]), the solution $x(t;\mathbf{p})$ can be analytically continued as a function of

the parameters $\mathbf{p}$ into the complex domain, and can be represented by so-called generalized polynomial chaos expansions with respect to multivariate tensorized, Legendre- and Chebyshev polynomials of $\mathbf{p}$ which *converge unconditionally on the possibly infinite-dimensional parameter space* $U = [-1,1]^{\mathbb{N}}$. To state these results (which are formulated and valid even for problems with infinitely many parameters indexed by $\mathbb{N}$, ie., for parameter sequences) we introduced in [1] the multi-index set

$$\mathfrak{F} = \{\nu \in \mathbb{N}_0^{\mathbb{N}} : |\operatorname{supp}\nu| < \infty\}\,, \quad \operatorname{supp}\nu = \{j \in \mathbb{N} : \nu_j \neq 0\}\ . \tag{2}$$

As any $\nu \in \mathfrak{F}$ has only finitely many nonzero entries, the definitions

$$\nu! = \prod_{j\in\mathbb{N}} \nu_j!\,, \quad |\nu| = \sum_{j\in\mathbb{N}} \nu_j\,, \quad \partial_{\mathbf{p}}^{\nu} = \frac{\partial^{|\nu|}}{\partial p_1^{\nu_1}\partial p_2^{\nu_2}\cdots}$$

for multi-factorials, the length of a multi-index ν and for the partial derivative of order ν are well-defined for $\nu \in \mathfrak{F}$.

At every $\mathbf{p} \in U \subset \mathbb{R}^{\mathbb{N}}$, the solution $x(t)$ of (1) can be represented by the formal Taylor expansion

$$x(t;\mathbf{p}) = \sum_{\nu\in\mathfrak{F}} T_\nu(t)\mathbf{p}^\nu, \tag{3}$$

where, for $\nu \in \mathfrak{F}$: $\mathbf{p}^\nu = p_1^{\nu_1}p_2^{\nu_2}...$ and

$$T_\nu(t) = \frac{1}{\nu!}\partial_{\mathbf{p}}^{\nu} x(t;\mathbf{p})|_{\mathbf{p}=0} \in C^{k+1}([0,T];\mathcal{S}), \quad \nu \in \mathfrak{F}\ .$$

The convergence of the formal Taylor expansion (3) is shown in [1] to be unconditional and pointwise in $[0,T]\times U$, i.e. we have pointwise convergence in U as a mapping taking values in the space $C^{k+1}([0,T];\mathcal{S})$, for any finite order k of differentiability with respect to the time variable t. Therefore, in particular, *the convergence theory for any standard timestepping scheme* for the approximate numerical solution of the parametric IVP (1) applies.

In our computational approach, we truncate the Taylor series (3) to a finite number of N terms, i.e. we consider

$$x_{\mathcal{M}_N}(t;\mathbf{p}) = \sum_{\nu\in\mathcal{M}_N} T_\nu(t)\mathbf{p}^\nu \tag{4}$$

with suitable index sets $\mathcal{M}_N \subset \mathfrak{F}$ of at most N indices. Evidently, the question is what the best/ optimal index set $\mathcal{M}_N$ is, second what the convergence rate for the error $x(t) - x_{\mathcal{M}_N}(t)$ is and, third, how such sets $\mathcal{M}_N$ could be spotted computationally. It was shown in [1] that there exist so-called *monotone* sequences of sparse index sets $\mathcal{M}_N \subset \mathfrak{F}$ (to which we will also refer as "sparsity models" for the parametric dependence of $x(t)$) which identify at most N "active" Taylor coefficients $T_\nu(t)$, $\nu \in \mathcal{M}_N$ in (3), which can be constructed such that the corresponding, finitely truncated parametric expansions (4) realize a convergence rate with respect to the number N of terms which equals the so-called *best N-term convergence rate.* Second, once such truncations have been selected, it will be necessary to solve the initial value problems by ODE solvers for approximation of the expansion coefficients $T_\nu(t)$ in (3).

A key role in our algorithms is played by index sets $\mathcal{M} \subset \mathfrak{F}$ that we refer to as *lower or monotone index sets.* This class of index sets was introduced in [2] in the context of adaptive, multivariate Taylor approximations of parametric elliptic partial differential equations. We now strengthen the N-term approximation properties of the Taylor series by constraining the admissible, nonempty index sets $\mathcal{M} \subset \mathfrak{F}$.

The notion of lower index sets is based on the following semi-ordering of $\mathfrak{F}$: for any two indices $\mu, \nu \in \mathfrak{F}$, we say that $\mu \leq \nu$ if and only if $\mu_j \leq \nu_j$ for all $j \geq 1$. We will also

say that $\mu < \nu$ if and only if $\mu \leq \nu$ for all $j \in \mathbb{N}$ and if $\mu_j < \nu_j$ for at least one value of j.

A sequence $(a_\nu)_{\nu \in \mathfrak{F}}$ of nonnegative real numbers is said to be *monotone decreasing* if and only if for all $\mu, \nu \in \mathfrak{F}$

$$\mu \leq \nu \Rightarrow a_\nu \leq a_\mu \ .$$

A nonempty set $\mathcal{M} \subset \mathfrak{F}$ is called *monotone* if and only if $\nu \in \mathcal{M}$ and $\mu \leq \nu \Rightarrow \mu \in \mathcal{M}$.

We now present the sparsity result [1, Theorem 5]. We consider the parametric IVP ODE (1) for parameter vectors $\mathbf{p} \in U = [-1,1]^{\mathbb{N}}$. We assume that there exist real numbers $R > 0$ and $0 < \kappa < 1$ with the following properties:

1. In (1) the vector field f depends on the parameter vector $\mathbf{p}$ in the affine fashion (1) with ϕ_j satisfying for some sparsity parameter $0 < \sigma < 1$

$$\begin{aligned} \left(\|\phi_j\|_{\ell\mathrm{Lip}_0(\mathcal{S},\mathrm{R})}\right)_{j\geq 1} &\in \ell^\sigma(\mathbb{N}) \ , \\ \left(\overline{\rho}_j \|\phi_j\|_{\ell\mathrm{Lip}_0(\mathcal{S},\mathrm{R})}\right)_{j\geq 1} &\in \ell^1(\mathbb{N}) \ , \end{aligned} \tag{5}$$

   where the polyradius $\overline{\rho}$ is given by $\overline{\rho}_j = \max\left(1, \frac{\delta}{4L_j(R)}\right)$ for some arbitrary fixed $\delta > 0$, and for $L_j(R) := \|\phi_j\|_{\ell\mathrm{Lip}_0(\mathcal{S},\mathrm{R})}$.

2. The initial data $x_0 \in C([0,T] \times U; \mathcal{S})$ satisfies a smallness condition (see [1, Theorem 5] for details).

$$\sup_{\mathbf{p}\in U} \|x_0(z)\| \leq (1-\kappa) R \exp(-TL(\overline{\rho}, R)/\kappa) \ . \tag{6}$$

Then the Taylor expansion (3) of the parametric solution $x(t;\mathbf{p})$ of (1) is σ-sparse in the following sense: for every $N \in \mathbb{N}$ there exists a monotone sparsity model $\mathcal{M}_N \subset \mathfrak{F}$ with $\#\mathcal{M}_N = N$ such that it holds, with rate $r = 1/\sigma - 1$,

$$\sup_{\mathbf{p}\in U} \left\| x(t;\mathbf{p}) - \sum_{\nu \in \mathcal{M}_N} T_\nu(t) \mathbf{p}^\nu \right\|_{L(\overline{\rho},R)/\kappa, T, \mathcal{S}} \leq C N^{-r}, \tag{7}$$

where the constant $C > 0$ is independent of n_p and of N, and where

$$\sum_{\nu \in \mathcal{M}_N} T_\nu(t) z^\nu \in \mathbb{P}_{\mathcal{M}_N}(U; C^{k+1}([0,T];\mathcal{S})) \tag{8}$$

with $\mathbb{P}_{\mathcal{M}}(U; C^{k+1}([0,T];\mathcal{S}))$ denoting the span of multivariate polynomials of $\mathbf{p} \in U$ with coefficients in the linear (Banach) space $C^{k+1}([0,T];\mathcal{S})$.

The actual, computational approximation of the parametric solution $x(t;\mathbf{p})$ family in the polynomial space $\mathbb{P}_{\mathcal{M}}(U; C^{k+1}([0,T];\mathcal{S}))$ can be constructed by *sparse interpolation* so that the (dimension-independent) convergence rate $1/\sigma - 1$ is realized by our proposed interpolation scheme. We now present some details of this scheme. Denoting by $(z^k)_{k\geq 0}$ a sequence of univariate interpolation points and by $(I^k)_{k\geq 0}$ the corresponding sequence of interpolation operators, the sparse interpolation operator is defined by

$$\mathcal{I}_{\mathcal{M}} = \sum_{\nu \in \mathcal{M}} \Delta_\nu = \sum_{\nu \in \mathcal{M}} \bigotimes_{j\geq 1} \Delta_{\nu_j} \tag{9}$$

with $\Delta_j = I^j - I^{j-1}$, $j \geq 0$. Under the assumption (5) and (6), and if the univariate sequence $(z^k)_{k\geq 0}$ is chosen such that the Lebesgue constant $\lambda_k \leq (k+1)^\theta$ for some $\theta \geq 1$, there exists a constant C and a nested sequence of monotone index sets $(\mathcal{M}_N)_{N\geq 1}$ with $\#\mathcal{M}_N = N$ such that

$$\sup_{\mathbf{p}\in U} \|x(t;\mathbf{p}) - \mathcal{I}_{\mathcal{M}_N} x(t;\mathbf{p})\|_{L(\overline{\rho},R)/\kappa, T, \mathcal{S}} \leq C N^{-r} \tag{10}$$

with $r = \frac{1}{\sigma} - 1$, (independent of number N of interpolation points and of the number of parameters), see [3]. Analogous to [3–5], we propose a deterministic, adaptive algorithm, which iteratively determines a nested sequence $(\mathcal{M}_N)_{N\geq 1}$ of monotone index sets to realize the rate (10).

The refinement strategy of the algorithm is based on a greedy-type approach aiming at localizing most profitable indices in a reduced neighborhood of the current index set defined by

$$\mathcal{N}(\mathcal{M}) := \{\nu \notin \mathcal{M} : \nu - e_j \in \mathcal{M}, \forall j \in \mathbb{I}_\nu \text{ and } \nu_j = 0\,, \forall j > j(\mathcal{M}) + 1\}$$

with $j(\mathcal{M}) = \max\{j : \nu_j > 0 \text{ for some } \nu \in \mathcal{M}\}$, $\mathbb{I}_\nu = \{j \in \mathbb{N} : \nu_j \neq 0\} \subset \mathbb{N}$. The estimated profit of an index $\nu \in \mathcal{N}(\mathcal{M}_k)$ in iteration k, denoted by g_ν, is measured according to its expected contribution to the approximation in terms of $g_\nu(\mathcal{M}_k; \Xi) = \max_{t\in\Xi} |x(t)(z^\nu) - \mathcal{I}_{\mathcal{M}_k} x(t)(z^\nu)|$ with z^ν denoting the corresponding interpolation point of the index ν and $\Xi \subset [0, T]$ is a suitable, finite subset, so that the dimension-adaptive index set is constructed recursively as follows.

Algorithm 1

function ASG
  Set $\mathcal{M}_0 = \{0\}\,, k = 0$
  Compute $\Delta_0(x(t)(\cdot))$ and the error indicator g_0
  Determine the set of reduced neighbors $\mathcal{N}(\mathcal{M}_1)$.
  Compute $\Delta_\nu(x(t)(\cdot))$ and the error indicator g_ν, $\forall \nu \in \mathcal{N}(\mathcal{M}_0)$.
  while $\max_{\nu\in\mathcal{N}(\mathcal{M}_k)} g_\nu > tol$ **do**
    Select ν from $\mathcal{N}(\mathcal{M}_k)$ with largest g_ν and set $\mathcal{M}_{k+1} = \mathcal{M}_k \cup \{\nu\}$.
    Determine the set of reduced neighbors $\mathcal{N}(\mathcal{M}_{k+1})$.
    Compute $\Delta_\nu(x(t)(\cdot))$ and $g_\nu\,, \forall \nu \in \mathcal{N}(\mathcal{M}_{k+1})$.
    Set $k = k + 1$.
  end while
end function

Details on the algorithm, which was originally proposed in the current version in [4], can also be found in [3, 6] and in the references therein. In the present case, after rescaling, the parameter domain $U = [-1, 1]^{n_p}$ (cp. Eqn [4] of the main text).

Furthermore, the sparsity results presented allow to design efficient, deterministic algorithms for identification of parameters in the underlying system from noisy measurements. The problem of computational inference of responses of uncertain systems in the presence of noisy observational data can be solved using a Bayesian approach, suitably generalized to (infinite-dimensional) function space settings in [7, 8]. In Bayesian prediction, the goal of computation is to evaluate numerically a mathematical expectation over all possible realizations of the unknown parameter sequence $\mathbf{p}$ conditional on given data $D \in \mathbb{R}^K$ stemming from K observations

$$y = h(\mathbf{p}) + \eta,$$

where the *uncertainty-to-observation map* $h : U \to \mathbb{R}^K$ is given by $h = \mathcal{O} \circ G$ with $\mathcal{O} :\to \mathbb{R}^K$ denoting a bounded, linear observation operator and with $G : U \to C^{k+1}([0, T]; \mathcal{S}))$ denoting the solution map. We assume throughout what follows that the Bayesian prior measure on the uncertain parameters $\mathbf{p}$ is the uniform measure denoted by $\mu_0(d\mathbf{p})$ and that the observational noise $\eta \in \mathbb{R}^K$ is an additive Gaussian random variable with law $\mathcal{N}(0, \Gamma)$ with some positive covariance matrix Γ that is assumed to be known. The present analysis can be generalized to the case of nonuniform, separable priors with compactly supported densities. In [8], it is shown that assuming the boundedness and continuity of $h(\mathbf{p})$, then $\mu^D(d\mathbf{p})$, the distribution of $\mathbf{p} \in U$ given D, is absolutely

continuous with respect to $\mu_0(d\mathbf{p})$, i.e.

$$\frac{d\mu^D}{d\mu_0}(\mathbf{p}) = \frac{1}{Z}\Theta(\mathbf{p})$$

with the parametric Bayesian likelihood Θ given by

$$\Theta(\mathbf{p}) = \exp\bigl(-\delta(\mathbf{p}; D)\bigr), \tag{11}$$

with $\delta(\mathbf{p}; D) := \frac{1}{2}|y - h(\mathbf{p})|_\Gamma^2$ and the normalization constant

$$Z = \int_U \Theta(\mathbf{p})\mu_0(d\mathbf{p})\ . \tag{12}$$

Note that this formulation is equivalent to the presentation in the main text, for example, with the normalization constant Z corresponding to the Bayesian evidence for the data.

In general, our aim is to compute the conditional expectation over all parameters $\mathbf{p}$ of a *quantity of interest* (QoI) Φ under the given noisy observational data and, in particular, we are interested in the case $\Phi = h(\mathbf{p})$, the states of the underlying system or higher moments of the states. To this end, Bayes' formula gives

$$\begin{aligned} \mathbb{E}[\Phi(\mathbf{p})] &= Z^{-1}\int_U \Phi(\mathbf{p})\mu^D(d\mathbf{p}) \\ &= Z^{-1}\int_U \Phi(\mathbf{p})\exp\bigl(-\delta(\mathbf{p}; D)\bigr)\mu_0(d\mathbf{p}) =: \frac{Z'}{Z} \end{aligned} \tag{13}$$

In [9], regularity and sparsity of the posterior density as a function of the parameter vector $\mathbf{p} \in U$ is analyzed. The results in [9], together with the sparsity analysis in [1], imply sparse approximations of the posterior with convergence rate r determined only by the model sparsity, but *independent of the model size.* These results are the basis for sparse, adaptive Smolyak quadrature algorithms to efficiently approximate the possibly infinite-dimensional integrals Z', Z defined in (13). Similar to the definition of the sparse interpolation operator in (9), we introduce the sparse quadrature operator for any finite monotone index set $\mathcal{M} \subset \mathfrak{F}$ by

$$\mathcal{Q}_{\mathcal{M}} = \sum_{\nu\in\mathcal{M}} \Delta_\nu = \sum_{\nu\in\mathcal{M}} \bigotimes_{j\geq 1} \Delta_{\nu_j} \tag{14}$$

with the quadrature difference operators $\Delta_\nu = \bigotimes_{j\geq 1} \Delta_{\nu_j}$, $\Delta_j = Q^j - Q^{j-1}$ and $(Q^k)_{k\geq 0}$ sequence of univariate quadrature formulas, see [6, 9] for details on the construction of the tensorized multivariate quadrature formulas.

Then it can be proven, under appropriate assumptions on the univariate quadrature formulas and on the forward problem, i.e. under the sparsity assumption (5) and the smallness condition (6), that there exists a sequence of finite, monotone index sets $(\mathcal{M}_N)_{N\geq 1}$ with $\#\mathcal{M}_N = N$ such that

$$|Z - \mathcal{Q}_{\mathcal{M}_N}(\Theta)| \leq C_Z N^{-r}\ , \tag{15}$$

and

$$\|Z' - \mathcal{Q}_{\mathcal{M}_N}(\Phi\Theta)\|_{L(\overline{\rho},R)/\kappa,T,\mathcal{S}} \leq C_{Z'} N^{-r} \tag{16}$$

with $r = \frac{1}{\sigma} - 1$. Analogously to the sparse interpolation, the construction of the monotone index sets $(\mathcal{M}_N)_{N\geq 1}$ is based on the greedy-type strategy summarized in Algorithm 1.

Note that the convergence rates presented are given with respect to the cardinality of the index set $\mathcal{M}$. Estimates of the work required for the evaluation of the adaptive

Smolyak approximation rely on the specific choice of the univariate interpolation and quadrature formulas. In [3], details on univariate interpolation formulas leading to the same convergence rates with respect to the number of interpolation points can be found. The quadrature case requires a slight adaption of the rate by a factor of $\log_2 3$, see [10, Proposition 1].

3 Models

To investigate the performance of the adaptive Smolyak approach, the proposed algorithm is applied to three models from the biochemical literature. The first model describes the uptake of glucose into cells of bakers' yeast *S. cerevisiae*, which is the first step of glycolysis. This model has 10 parameters that were assumed to be uncertain. Due to the moderate size, this model gives an opportunity to verify that our method is correctly implemented by comparison to Monte-Carlo simulations.

The second model describes the short-term response of the epidermal growth factor receptor (EGFR) pathway for stimulation with EGF. This model has 50 parameters, which is a typical number of parameters for models of cell signaling in the literature, but constitutes a substantial challenge for any known computational method for statistical inference due to the curse of dimensionality.

The third model describes the coupling of signaling pathways in mammalian cells. With 227 parameters, it is to our knowledge one of the largest dynamical models reported in the literature.

3.1 Model 1 - Glucose Transport in Yeast

The ODEs that describe the system dynamics are based on mass-action kinetics and take the form:

$$\frac{dx^e_{Glc}}{dt} = -k_1 x^e_E x^e_{Glc} + k_{-1} x^e_{E-Glc} \tag{17a}$$

$$\frac{dx^i_{Glc}}{dt} = -k_2 x^i_E x^i_{Glc} + k_{-2} x^i_{E-Glc} \tag{17b}$$

$$\frac{dx^i_{E-G6P}}{dt} = k_4 x^i_E x^i_{G6P} - k_{-4} x^i_{E-G6P} \tag{17c}$$

$$\frac{dx^i_{E-Glc-G6P}}{dt} = k_3 x^i_{E-Glc} x^i_{G6P} - k_{-3} x^i_{E-Glc-G6P} \tag{17d}$$

$$\frac{dx^i_{G6P}}{dt} = -k_3 x^i_{E-Glc} x^i_{G6P} + k_{-3} x^i_{E-Glc-G6P} - k_4 x^i_E x^i_{G6P} + k_{-4} x^i_{E-G6P} \tag{17e}$$

$$\frac{dx^e_{E-Glc}}{dt} = \alpha(x^i_{E-Glc} - x^e_{E-Glc}) + k_1 x^e_E x^e_{Glc} - k_{-1} x^e_{E-Glc} \tag{17f}$$

$$\frac{dx^i_{E-Glc}}{dt} = \alpha(x^e_{E-Glc} - x^i_{E-Glc}) - k_3 x^i_{E-Glc} x^i_{G6P} + k_{-3} x^i_{E-Glc-G6P} + k_2 x^i_E x^i_{Glc} - k_{-2} x^i_{E-Glc} \tag{17g}$$

$$\frac{dx^e_E}{dt} = \beta(x^i_E - x^e_E) - k_1 x^e_E x^e_{Glc} + k_{-1} x^e_{E-Glc} \tag{17h}$$

$$\frac{dx^i_E}{dt} = \beta(x^e_E - x^i_E) - k_4 x^i_E x^i_{G6P} + k_{-4} x^i_{E-G6P} - k_2 x^i_E x^i_{Glc} + k_{-2} x^i_{E-Glc} \tag{17i}$$

where the nine state variables correspond to concentrations of the following chemical species: external glucose x^e_{Glc}, internal glucose x^i_{Glc}, internal G6P-bound carrier

x^i_{E-G6P}, internal glucose and G6P-bound carrier $x^i_{E-Glc-G6P}$, internal G6P x^i_{G6P}, external glucose-bound carrier x^e_{E-Glc}, internal glucose-bound carrier x^i_{E-Glc}, external free carrier x^e_E, and internal free carrier x^i_E. The model has 10 rate parameters: k_1, k_{-1}, k_2, k_{-2}, k_3, k_{-3}, k_4, k_{-4}, α, β. We also need to specify at least three initial conditions for (external) glucose, G6P, and for carrier E, if we assume that the concentrations of the other chemical species are initially negligible.

This model was originally introduced in a reduced form in Rizzi et al. [11], and a derivation of the reduced model from Eq. (17) is presented in [12]. For this article, the rate parameters have been chosen as in [11] to reflect the assumptions used to derive the reduced model (e.g., the time scales of reactions resulting in quasi-steady state relations). The artificial observation data used in the numerical experiments is generated from a randomly chosen reference parameter ($10^{\pm 0.25}$ variations around the nominal point). Observations are assumed to be available for the first state (external glucose) at time points $20.0, 30.0, 60.0$ and for the first, second and fifth state at time point 30.0. The noise in the measurements is given by a normal distribution with variance 0.1^2.

3.2 Model 2 - Epidermal growth factor receptor (EGFR) signaling

This model describes the short-term (up two minutes after stimulation) response of the epidermal growth factor receptor (EGFR) pathway upon stimulation with EGF [13]. The model was created to explain why the concentration of phosphorylated EGFR, phospholipase C-γ (PLC$_\gamma$), and the total concentration of Grb2-EGFR complexes, peak after about half a minute and then return to low levels within a few minutes upon EGF stimulation. Specifically, it was proposed that the peak in EGFR phosphorylation could be explained by EGFR being protected from phosphatases when bound to target molecules (Grb2, Shc, or PLC$_\gamma$), rather than by a rapid activation of (tyrosine) phosphatases.

The model has 50 kinetic parameters, which were determined from previous experimental results in the literature. The one-dimensional sensitivity analysis in [13] demonstrated that most parameters could be individually varied without significant changes in the pathway response. However, changing all parameters simultaneously may result in a "marked change in the response dynamics", although it was also pointed out that a multiplication of all estimated parameters values by 2 only results in a scaling in time. It was also noted that the model is sensitive to the relative concentrations of signaling proteins. By applying our method it is possible to investigate the model response to variations in any combination of parameter values around the estimated optimal parameter point. Within the inverse setting, we use artificial data, generated from a randomly chosen parameter value in the range of $10^{\pm 0.1}$ and $10^{\pm 0.25}$ around nominal (assuming a uniform prior distribution) and perturbed by normally distributed noise. For all numerical experiments presented below, the first state of the model is observed at discrete time points $20.0, 30.0, 60.0$.

3.3 Model 3 - Coupled signaling pathways

This model describes the EGF (and heregulin) activated response in, and interaction of, a number of signaling pathways in mammalian (rat) cells. Components of the signaling network that can be simulated with this model are the mammalian ErbB signaling pathways and the MAPK and Akt cascades.

The model exists in two forms: with and without rules for time-dependent assignments of parameter values. We used the model without rules, downloaded from the supplementary material of [14], since only this model is directly compatible with our odeSD solver [15]. However, as pointed out by Chen et al. the difference between the models is small, although the model without rules cannot be simulated in the "pre-

incubation" phase. We decided to keep the 500 initial conditions for the state variables of the model fixed to the values specified in the model, and to explore the impact of all the 227 model parameters in the dimension-adaptive algorithm.

Detailed computational results of 20 runs of the optimiziation algorithm by Chen et al. are provided in the supplementary material in [14], and 48 parameters and 9 initial conditions were calibrated to different values (in the A431 cell line). The remaining model parameters were either fixed to the values predefined in the model, or estimated to very similar values in all the runs of the optimization algorithm (numerically equivalent for the precision provided in the file: `Parameters_A431_20models.mat` in the supplementary material in [14]). Note that the model response variables should be sensitive to the parameters that are always estimated to similar values.

Our computations of the local sensitivities were made for the parameter point that is incorporated in the following model file (supplementary material of [14]):
`ErbB-Chen_et_al_2008-A431-norules.xml`
which was found in the folder:
`inline-supplementary-material-2\_dataset\Chen` et al - Model files
in the zip-file:
`msb200874-sup-0002.zip`
on the website:
`http://msb.embopress.org/content/5/1/239#sec-26`
Artificial data was generated by simulating the model for this parameter point, and adding Gaussian noise to the response variables (Akt, Erk, and ErbB1) in the model corresponding to entities that can be experimentally measured. The reference values used to generate the artificial data are randomly chosen in the parameter box $\mathbf{p}_0 \pm 0.01\mathbf{p}_0$. The response variables Akt, Erk and ErbB1 are observed at time instants $75.0, 100.0, 150.0$ and $50.0, 75.0, 100.0, 150.0$, respectively. The noise in the data is modeled by a normal distribution with variance 0.1^2.

4 Numerical Experiments

In the following section, we present numerical results of the proposed method for all three models introduced in Sec. S3. We compare the results and the performance of the adaptive Smolyak algorithm to state-of-the art methods (first-order sensitivity approach and MCMC methods) and discuss the improvements achieved as well as our method's limitations.

4.1 Model 1

4.1.1 Comparison between the adaptive Smolyak interpolation and a first-order sensitivity approach

In systems biology, common methods to analyze the system behavior with respect to changes in the parameter space are local methods based on first-order-sensitivity information. In the following, we will compare the proposed adaptive Smolyak interpolation with a first-order approach approximating the solution $x(t; p)$ by $\tilde{x}(t; \mathbf{p}) = x(t; \mathbf{p}_0) + J_{\mathbf{p}}(t)(\mathbf{p} - \mathbf{p}_0)$, where $J_{\mathbf{p}}$ denotes the Jacobian with respect to the parameters at the nominal point $\mathbf{p}_0$. The quantities of interest are the first, second and fifth state in Eq. (17) (external and internal glucose and internal G6P) at time instants $[0, 12, 20, 30, 60, 120, 200, 300, 400, 500, 1000]$ for parameter variations in the range of $\pm 0.25\mathbf{p}_0$.

The sensitivity profile, which quantifies the maximum (absolute value of the) sensitivity over states and time with respect to the parameters, is shown in Fig. S1. The

sensitivity index σ, defined by

$$\sigma := \max_{\mathbf{p}}\{\max_{x_i(t_j), i=1,\dots,n_x, j=1,\dots,n_t} (\partial_{\mathbf{p}} x_i)(t_j)\}\,, \tag{18}$$

with n_x number of states and n_t number of discrete time points, equals 0.1866 for model 1.

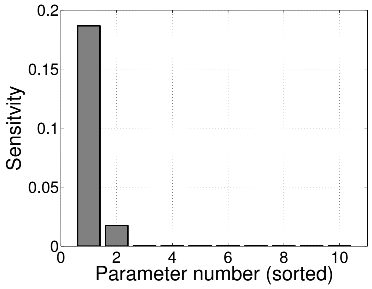

Figure S1. Sensitivity profile of the first model: absolute value of the sensitivities with respect to the parameters at the nominal point $\mathbf{p}_0$ sorted in descending order with respect to maximum over states $x_1, \dots, x_9$ and time instants $[0.0, 12.0, 20.0, 30.0, 60.0, 120.0, 200.0, 300.0, 400.0, 500.0., 1000.0]$ of each parameter.

The sparse interpolation operator (9) is constructed using Clenshaw-Curtis points or projected Leja sequences defined as follows:

- Clenshaw-Curtis (CC),

$$z_j^k = -\cos\left(\frac{\pi j}{n_k - 1}\right),\, j = 0, \dots, n_k - 1, \text{if } n_k > 1 \text{ and}$$
$$z_0^k = 0\,, \text{if } n_k = 1$$

  with $n_0 = 1$ and $n_k = 2^k + 1$, for $k \geq 1$

- $\mathfrak{R}$-Leja sequence (RL),
  projection on $[-1, 1]$ of a Leja sequence for the complex unit disk initiated at i

$$z_0^k = 0\,, z_1^k = 1\,, z_2^k = -1\,, \text{if } j = 0, 1, 2 \text{ and}$$
$$z_j^k = \mathfrak{R}(\hat{z}), \text{ with } \hat{z} = \arg\max_{|z|\leq 1} \prod_{l=1}^{j-1} |z - z_l^k|\,, j = 3, \dots, n_k, \text{if } j \text{ odd}\,,$$
$$z_j^k = -z_{j-1}^k\,, j = 3, \dots, n_k, \text{if } j \text{ even}\,,$$

  with $n_k = 2 \cdot k + 1$, for $k \geq 0$.

Fig. S2 displays the estimated (absolute) interpolation error in terms of the number of indices in the adaptively constructed index set and in terms of the number of ODE solves needed. We observe that both interpolation sequences lead to an almost identical convergence behavior of the Smolyak algorithm with convergence rate 1 (with respect to the number of ODE solves needed). Note that, in the following, Λ denotes the monotone index set computed by the adaptive algorithm given in 1.

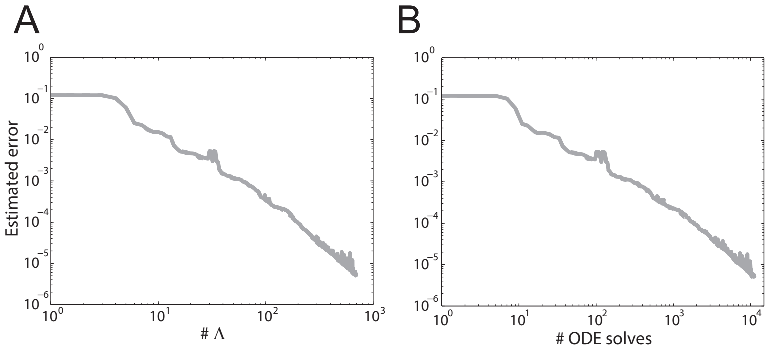

Figure S2. Estimated L^∞ (absolute) error curves of the interpolation of the states $1, 2, 5$ with respect to the cardinality of the index set Λ_N based on the sequences CC and RL (left) and with respect to the number of ODE solves needed (right), variations of $\pm 0.25\mathbf{p}_0$, first model. The solid line corresponds to the interpolation based on CC points, the dashed line to the interpolation based on Leja points. Both interpolation operators show an almost identical approximation behavior, so that the two lines are on top of each other.

In order to compare the proposed dimension-adaptive sparse grid approach with the first-order approximation, we evaluate both approximations at three randomly chosen realizations of the parameters, shown in Figs. S3-S5.

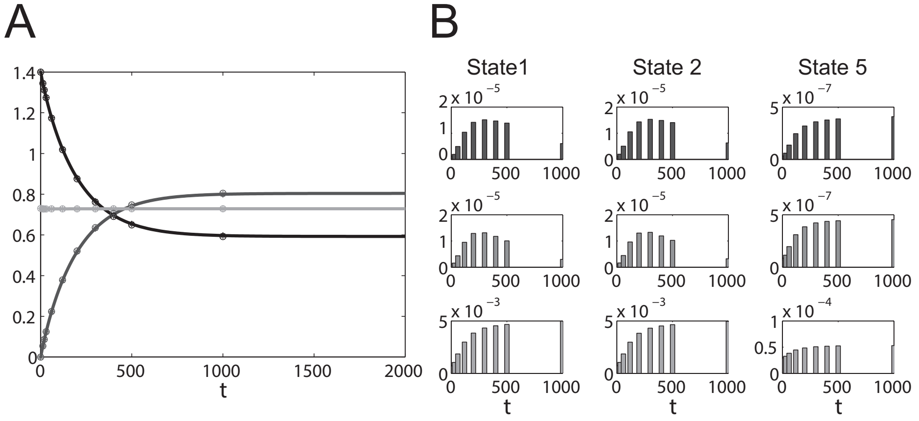

Figure S3. Methods comparison for forward simulation of model 1, realization 1. (**A**) Comparison of the states approximated by the adaptive sparse grid based on CC and RL interpolation nodes, the first-order sensitivity approximation and the solution of the underlying ODE at a randomly chosen realization of the parameter (variations of $\pm 0.25\mathbf{p}_0$). (**B**) Resulting errors, namely (absolute) errors from the adaptive interpolation based on CC points (top row), from the interpolation based on Leja points (middle row) and from a first-order sensitivity approximation (bottom row).

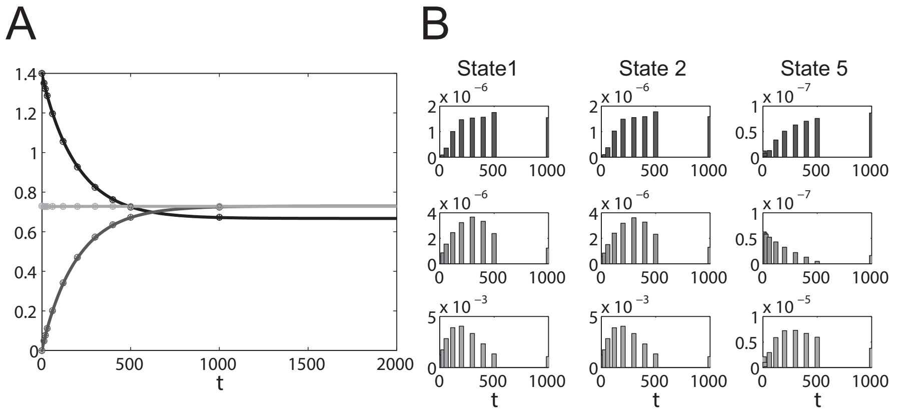

Figure S4. Methods comparison for forward simulation of model 1, realization 2 (see caption of Fig. S3 for details).

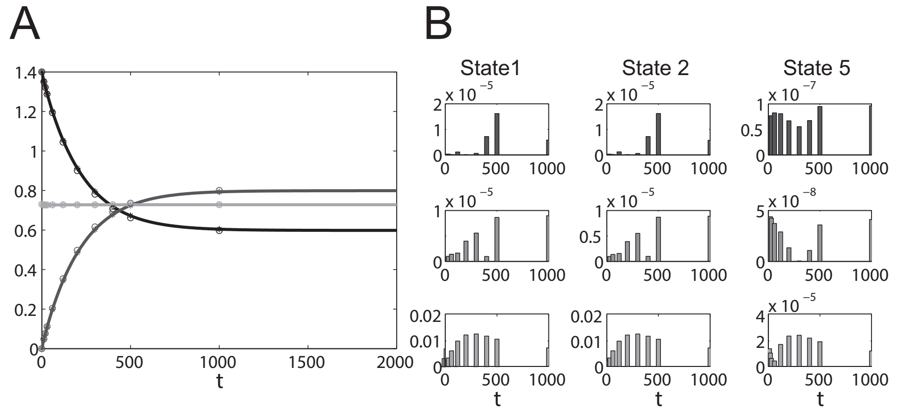

Figure S5. Methods comparison for forward simulation of model 1, realization 3 (see caption of Fig. S3 for details).

We first notice that the Smolyak interpolation based on CC and RL points leads to errors in the range of 10^{-5}, as predicted by the error estimator. The approximation error of the first-order approach is in the range of 10^{-3}, which means a loss in accuracy of two orders of magnitude compared to the sparse grid interpolation. Increased accuracy can be crucial to suppress numerical errors in the analysis of system quantities and calibration to measurements. The adaptive sparse interpolation provides an efficient way to approximate the solution on the entire parameter space and thus, in contrast to local methods, allows to consider larger domains of parameter variations.

4.1.2 Bayesian inverse problem

We also estimated error curves for Bayesian inference with the Smolyak based method, which are shown for the normalization constant Z in Fig. S6, and for the quantity Z' of the first moment of the six non-measurable state variables in Fig. S7, given observations of the first state variable (external glucose). The first and second moments of the six non-observable state variables over time are shown in Fig. S8 and Fig. S9, respectively.

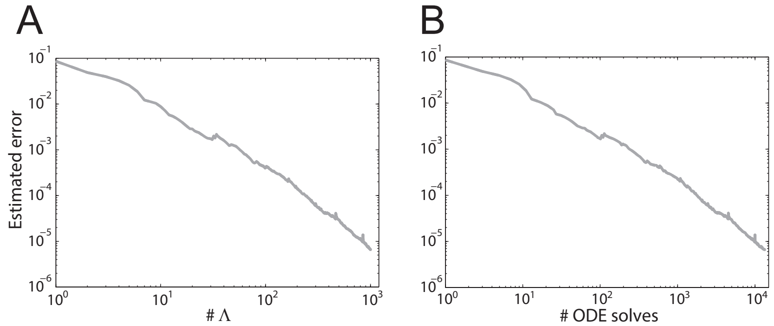

Figure S6. Estimated (absolute) error curves of the normalization constant Z with respect to the cardinality of the index set Λ_N based on the sequences CC and RL (**A**) and with respect to the number of ODE solves needed (**B**), uniform distribution of the parameters, variations of $\mathbf{p}_0 \cdot 10^{\pm 0.25}$, $\eta \sim \mathcal{N}(0, 0.1^2)$ and 3 observations of state 1,2,5 at $t_{obs} = [30.0]$, first model. The solid line corresponds to the interpolation based on CC points, the dashed line to the interpolation based on Leja points. Both interpolation operators show an almost identical approximation behavior, so that the two lines are on top of each other.

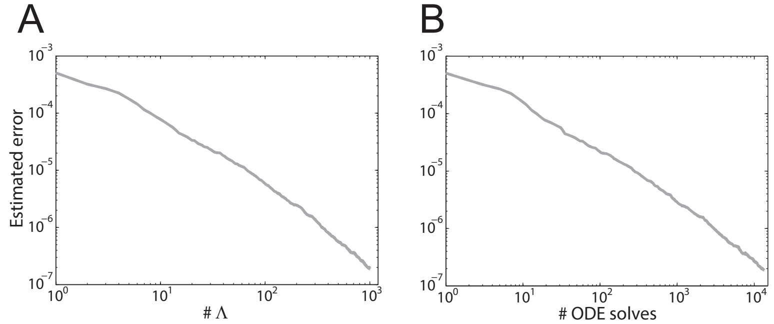

Figure S7. Estimated (absolute) error curves of the first moment of state $3, 4, 6, 7, 8, 9$ with respect to the cardinality of the index set Λ_N based on the sequences CC and RL (left) and with respect to the number of ODE solves needed (right), uniform distribution of the parameters, variations of $\mathbf{p}_0 \cdot 10^{\pm 0.25}$, $\eta \sim \mathcal{N}(0, 0.1^2)$ and 3 observations of states 1,2,5 at $t_{obs} = [30.0]$, first model. The solid line corresponds to the interpolation based on CC points, the dashed line to the interpolation based on Leja points, both lines are on top of each other.

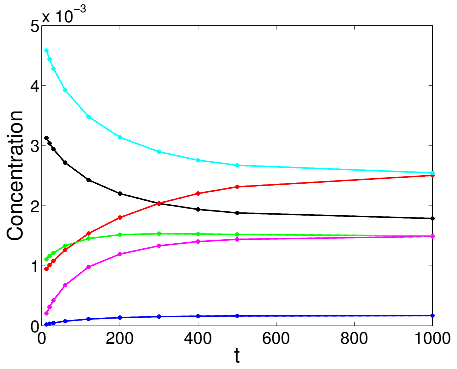

Figure S8. Conditional expectations of the first moment of state 3 (black), 4 (blue), 6 (green), 7 (pink), 8 (red), 9 (light blue) at
$t = [12.0, 20.0, 30.0, 60.0, 120.0, 200.0, 300.0, 400.0, 500.0.1000.0]$ based on the sequences CC and RL, uniform distribution of the parameters, variations of $\mathbf{p}_0 \cdot 10^{\pm 0.25}$, $\eta \sim \mathcal{N}(0, 0.1^2)$ and 3 observations of states 1,2,5 at $t_{obs} = [30.0]$, first model.

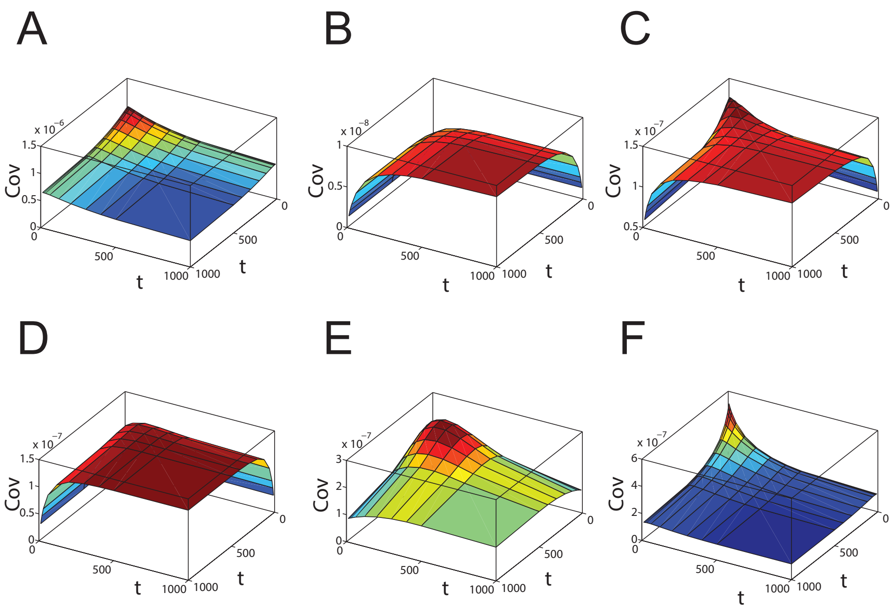

Figure S9. Conditional expectations of the covariance of state 3 (A), 4 (B), 6 (C), 7 (D), 8 (E), 9 (F) at $t = [12.0, 20.0, 30.0, 60.0, 120.0, 200.0, 300.0, 400.0, 500.0.1000.0]$ based on the sequences CC and RL, uniform distribution of the parameters, variations of $\mathbf{p}_0 \cdot 10^{\pm 0.25}$, $\eta \sim \mathcal{N}(0, 0.1^2)$ and 3 observations of states $1, 2, 5$ at $t_{obs} = [30.0]$, first model.

4.1.3 Comparison between Monte Carlo and the adaptive Smolyak approach

To analyze the glucose transport model in section 3.1, we used a Metropolis Hastings Markov Chain Monte Carlo (MH-MCMC) algorithm [16], and the results are then compared to those of the Smolyak approach.

Assume that we have the following (artificial) observations of the first state variable in the model x^e_{Glc} (external glucose) at time instants: $x^e_{Glc}(t = 20) = 1.4695$, $x^e_{Glc}(t = 30) = 1.2386$, $x^e_{Glc}(t = 60) = 0.9625$. We also assume that the standard deviation for

the measurement noise is known and equal to 0.1 for all three time instants. We explore the parameter space in the interval $[\mathbf{p}_0 \cdot 10^{-0.25}, \mathbf{p}_0 \cdot 10^{+0.25}]$, where $\mathbf{p}_0$ is the nominal point of model 1. The proposal distribution that we use for MH-MCMC is a uniform distribution on the interval of considered parameter values (± 0.25, around the nominal parameter point in log-space) to compute estimates of the model entities. This makes a burn-in period (with discarded parameters) unnecessary, since the proposal distribution remains the same along the chain. However, we also tested the performance of proposal distributions with narrower distributions, for which the results were similar in terms of the convergence rate (data not shown). This strategy is of course only feasible in the case of artificial data, since in general, the unknown data is not at hand. Hence, the burn-in period is considerable and leads to a significant increase in the overall number of forward simulations needed to sample from the posterior.

To investigate the convergence rate of the MH-MCMC approach we generated a sequence of parameter points from the posterior distribution, and computed the posterior first moment of the state variables of interest based on all drawn parameter points. A sufficient number of parameter points was drawn (100.000 points) to ensure that the drawn parameter points could be used to compute the first moments to a sufficient numerical precision. We then simulated a second, independent sequence of parameter points from the posterior and estimated the distance to convergence for the points along this sequence, with the first chain as a reference. We used an empirical measure E_k for convergence, after k samples have been drawn from the prior, which is given by:

$$E_k = \max_{i=3,4,6,7,8,9} \max_{j=t_1,\ldots,t_{10}} |y_{ijk} - \hat{y}_{ijK}| \tag{19}$$

where the index i refers to the six non-observed state variables, $t_1, \ldots, t_{10}$ denote the discrete time instants, $y_{ijk} = \frac{1}{k}\sum_{l=1}^{k} y_{ijl}, k = 1, \ldots, K$ is the mean of state i at time instant j for the first k points of the Markov chain, and $\hat{y}_{ijK}$ is the estimated averaged over K points from a separate Markov chain.

The results are presented in Fig. 2E. Monte Carlo has a convergence rate of around 0.5 (in mean square with respect to the prior measure) as expected, which should be compared to the rate of around $2.6/4.1 \approx 0.65$ (in the maximum norm, ie., in the worst-case setting) for the adaptive Smolyak approach. However, note that the convergence rate of Smolyak is close to that of MH-MCMC for a couple of hundred ODE solves, before transitioning to a new (higher) asymptotic convergence rate which is stable (at around 1.2) after the first few thousand ODE solves. The convergence rate of the adaptive Smolyak approach is higher than for the MH-MCMC approach, indicating a significant improvement. However, the adaptive Smolyak approach does not seem to reach its full potential in this case since the parametric response of the model does not appear to be sparse (the model response appears to be equally sensitive to most parameters). Finally, we note that other numerical experiments, with different response variables and observation time points, show better convergence rates (> 1), cf. Fig. S6 and Fig. S7.

To verify that the adaptive Smolyak algorithm is correctly implemented we computed the first two moments of the state variables for the posterior with MH-MCMC, and compared those to the moments computed with adaptive Smolyak. The results for the first moment are shown in Fig. S10, where we observe an excellent agreement. The second moment of the state variables computed with MH-MCMC is presented as the covariance over time, and is shown in Fig. S11. Also here the results of MH-MCMC agree with those of adaptive Smolyak. We therefore conclude that the adaptive Smolyak method is correctly implemented, and that it converges faster than MH-MCMC, although the rate of convergence is strongly dependent on the sparsity of the model (cf. Fig. 2E).

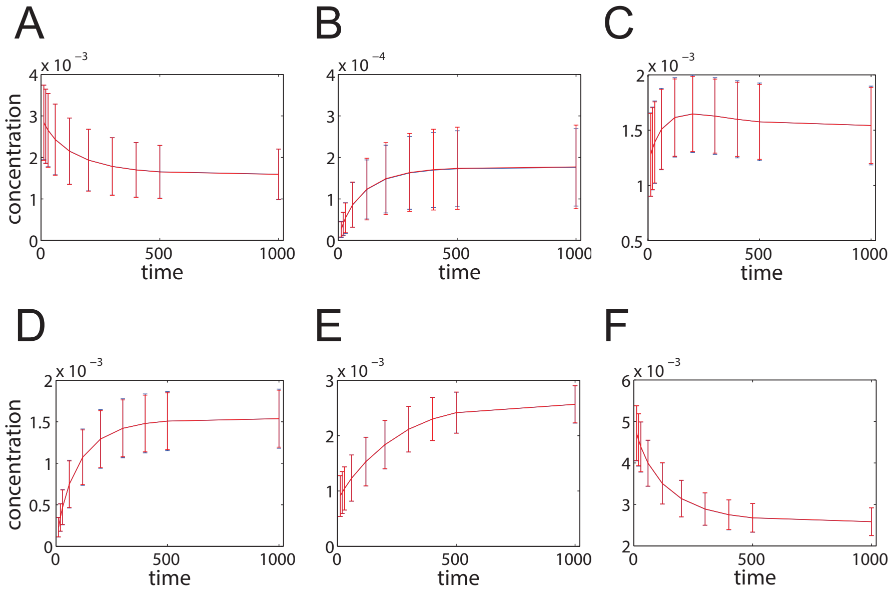

Figure S10. First moment of the posterior parameter distribution. Comparison of the first moment of the posterior for the unobserved state variables (A: x^i_{E-G6P}, B: $x^i_{E-Glc-G6P}$, C: x^e_{E-Glc}, D: x^i_{E-Glc}, E: x^e_E, F: x^i_E) computed with the Monte Carlo (blue) and adaptive Smolyak approach (red), variations of $\mathbf{p}_0 \cdot 10^{\pm 0.25}$, $\eta \sim \mathcal{N}(0, 0.1^2)$ and 3 observations of state 1 at $t_{obs} = [20.0, 30.0, 60.0]$, first model.

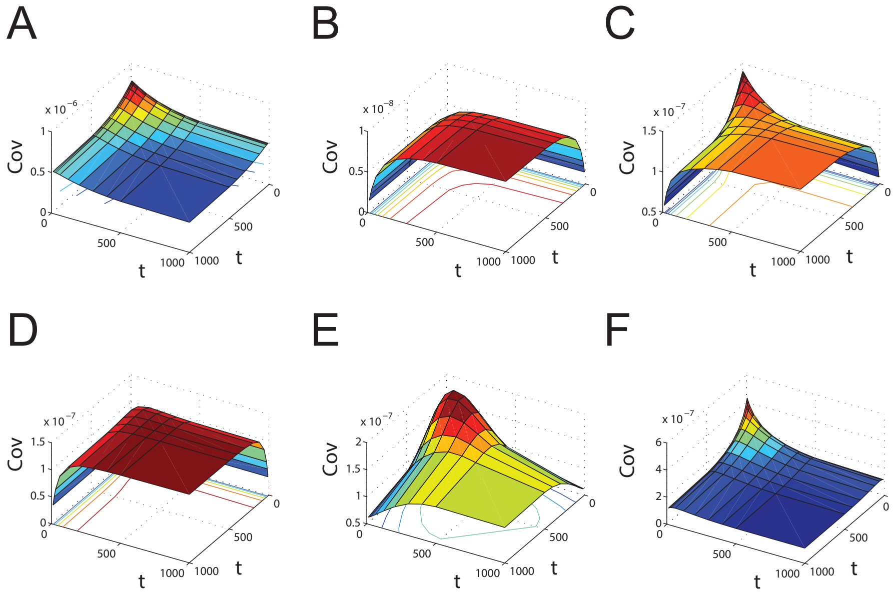

Figure S11. Second moment of the posterior parameter distribution for Monte Carlo. For each state variable (A: x^i_{E-G6P}, B: $x^i_{E-Glc-G6P}$, C: x^e_{E-Glc}, D: x^i_{E-Glc}, E: x^e_E, F: x^i_E) the second moment is presented as the covariance at several time instants, variations of $\mathbf{p}_0 \cdot 10^{\pm 0.25}$, $\eta \sim \mathcal{N}(0, 0.1^2)$ and 3 observations of state 1 at $t_{obs} = [20.0, 30.0, 60]$, first model.

4.2 Model 2

4.2.1 Comparison between the adaptive Smolyak interpolation and a first-order sensitivity approach

We investigate the interpolation behavior of the Smolyak algorithm in the 50-dimensional parameter space for variations in the range of $\pm 0.25\mathbf{p}_0$ at time instants $[0.0, 12.0, 20.0, 30.0, 60.0, 120.0]$.

A sensitivity analysis of the model, the sensitivity profile is displayed in Fig. S12, gives the sensitivity index σ, defined by (18) with $n_x = 23,\ n_t = 6,\ \sigma = 5.8058e3$.

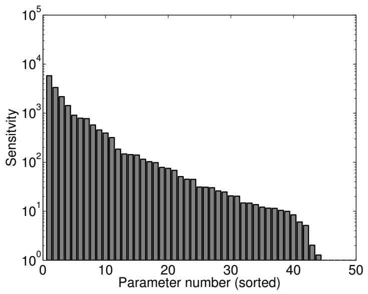

Figure S12. Sensitivity profile of the second model: absolute value of the sensitivities with respect to the parameters at the nominal point $\mathbf{p}_0$ sorted in descending order with respect to maximum over states $x_1, \ldots, x_{23}$ and time points $[0.0, 12., 20.0, 30.0, 60.0, 120.0]$ of each parameter.

The sparse Smolyak interpolation approach gives the estimated error curves shown in Fig. 3A, which suggest a convergence rate of 0.75 with respect to the number of ODE solves needed. As discussed in section 2, the convergence rate is determined by the sparsity of the underlying problem.

Taking a look at the index set computed by the adaptive algorithm, we notice that the interpolation of the states requires an almost isotropic refinement (with interpolation formulas of order 1 and 2), cf. Fig. 3B, limiting the convergence rate.

Next, we compare the proposed approach with the first-order approximation by measuring the approximation error at three randomly chosen realizations of the 50 parameters, displayed in Fig. 3C, Fig. S13 and Fig. S14.

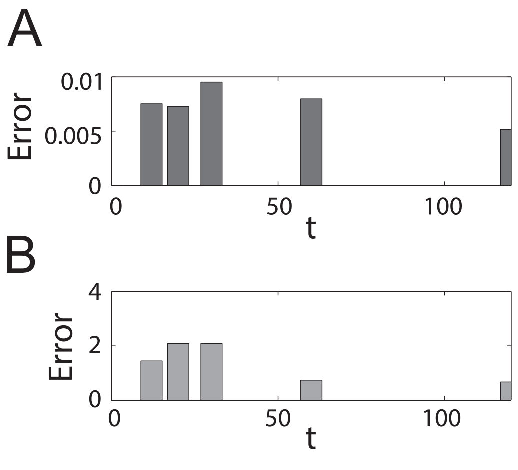

Figure S13. Comparison of the (absolute) errors or the adaptive sparse grid based on CC interpolation nodes (above) and the first-order sensitivity approximation (below), variations of $\pm 0.25\mathbf{p}_0$, 2nd realization, second model.

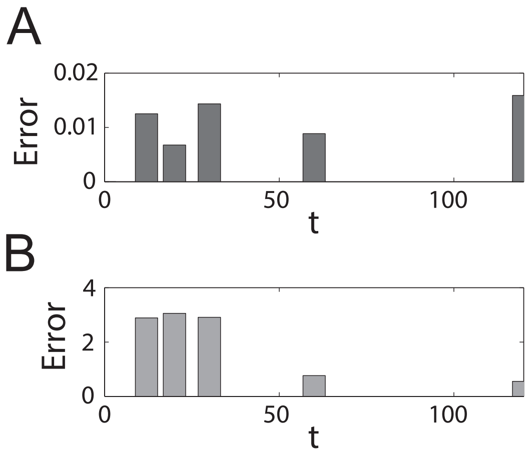

Figure S14. Comparison of the (absolute) errors for the adaptive sparse grid based on CC interpolation nodes (above) and the first-order sensitivity approximation (below), variations of $\pm 0.25\mathbf{p}_0$, 3rd realization, second model.

As already observed in the previous numerical example, the local method cannot capture the global behavior of the states and leads to an error of order 1, whereas the Smolyak approximation shows an error of the estimated order 10^{-2}.

4.2.2 Analysis of 'sloppy' parameter sensitivities

In [17], changes in model behavior upon parametric perturbations are measured by the average squared change in the model states

$$\chi^2(p) = \frac{1}{n_x} \sum_{k=1}^{n_x} \frac{1}{n_t} \sum_{l=1}^{n_t} \left(\frac{x_k(t_l, p) - x_k(t_l, p_0)}{\sigma_k} \right)^2 , \tag{20}$$

where p_0 denotes the nominal value of the parameters, n_x denotes the number of states, n_t is the number of time points considered in the model and σ_k is defined as the maximum of state k over all (discrete) time points, i.e. $\sigma_k = \max_{t \in \{t_0, \ldots, t_{n_t - 1}\}} x_k(t, p_0)$.

The Hessian matrix of χ^2 is computed to analyze the sensitivity of the models to parameter variations. Since in our models, we already reparametrized the parameter space, such that the parameter variations are in the range of $[-1, 1]$, we do not consider derivatives w.r.t. $\log p$ as suggested in [17] to overcome scaling issues. The Hessian matrix

$$H_{jk} = \frac{\mathrm{d}\chi^2}{\mathrm{d}p_j \, \mathrm{d}p_k} \tag{21}$$

is then approximated using finite differences, i.e. we compute an approximation $\tilde{H}$ by

$$\begin{aligned} \tilde{H}_{jk} &= \frac{\chi^2(p_0 + \epsilon e_j + \epsilon e_k) - \chi^2(p_0 + \epsilon e_j) - \chi^2(p_0 + \epsilon e_k) + \chi^2(p_0)}{\epsilon^2} \\ &= \frac{\chi^2(p_0 + \epsilon e_j + \epsilon e_k) - \chi^2(p_0 + \epsilon e_j) - \chi^2(p_0 + \epsilon e_k)}{\epsilon^2} , \end{aligned}$$

since $\chi^2(p_0) = 0$. The Hessian approximation is computed with a finite difference step size $\epsilon = 10^{-6}$.

4.2.3 Bayesian inverse problem

In our next numerical experiment, we are interested in the inverse problem, i.e. the goal of computation is the conditional expectation of a quantity of interest, state 1,

with respect to noisy measurements. The 50 parameters are assumed to be uniformly distributed with variations in the log space of the range $\mathbf{p}_0 \cdot 10^{\pm 0.1}$. The noise of the (synthetic) measurement data is modeled by a Gaussian random variable with zero mean and standard deviation 1. We observe a convergence order of almost 1 with respect to the number of ODE solves required for the approximation of the normalization constant and of the quantity Z' defined by (13), see Fig. S15 and Fig. S16. Enlarging the parameter variations to $\mathbf{p}_0 \cdot 10^{\pm 0.25}$ (Fig. S17), we observe a deterioration of the convergence rate, caused by the increasing nonlinearity of the underlying model. However, compared to the state of the art Monte-Carlo methods, the convergence rate r is only limited by the intrinsic sparsity of parametric system response, with, in addition, maximum-norm (or worst-case) error bounds thus enabling computational uncertainty quantification and parameter identification also for complex reaction networks on high-dimensional parameter spaces.

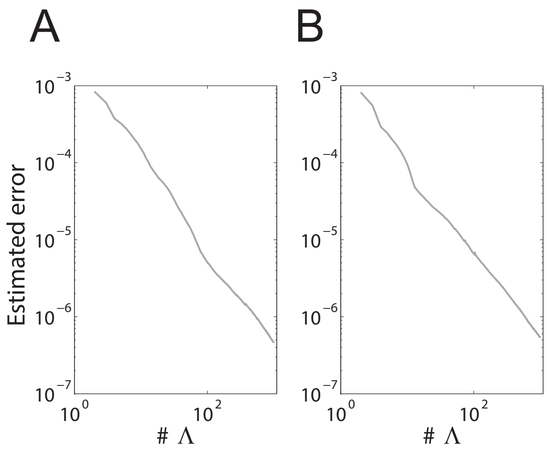

Figure S15. Estimated (absolute) error curves of the approximations for the normalization constant Z (left) and the quantity Z' (right) with respect to the cardinality of the index set Λ_N based on the sequences CC, uniform distribution of the uncertain parameters, variations of $\mathbf{p}_0 \cdot 10^{\pm 0.1}$, $\eta \sim \mathcal{N}(0, 1.0)$ and 3 observations of state 1 at $t_{obs} = [20.0, 30.0, 60.0]$, second model.

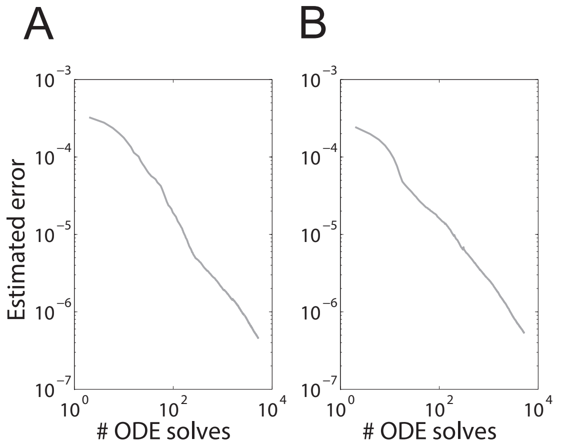

Figure S16. Estimated (absolute) error curves of the approximations for the normalization constant Z (left) and the quantity Z' (right) with respect to the number of ODE solves needed based on the sequences CC, uniform distribution of the uncertain parameters, variations of $\mathbf{p}_0 \cdot 10^{\pm 0.1}$, $\eta \sim \mathcal{N}(0, 1.0)$ and 3 observations of state 1 at $t_{obs} = [20.0, 30.0, 60.0]$, second model.

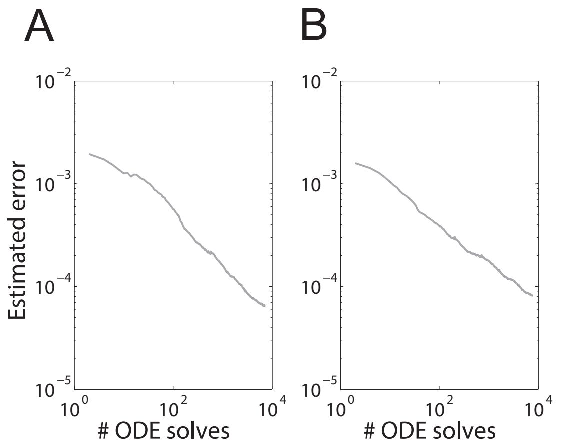

Figure S17. Estimated (absolute) error curves of the approximations for the normalization constant Z (left) and the quantity Z' (right) with respect to the number of ODE solves needed based on the sequences CC, uniform distribution of the uncertain parameters, variations of $\mathbf{p}_0 \cdot 10^{\pm 0.25}$, $\eta \sim \mathcal{N}(0, 1.0)$ and 3 observations of state 1 at $t_{obs} = [20.0, 30.0, 60.0]$, second model.

The numerical experiment discussed above clearly demonstrates the potential of the proposed approach, especially for problems which depend on high-dimensional parameter spaces. The convergence rates and approximation error could be significantly improved compared to the state of the art methods. Limitations of the adaptive Smolyak approach arise by increasing the parameter domain leading to a highly nonlinear behavior of the underlying model, which cannot be captured properly by global approximations. The deterioration of the convergence rate can be also observed for standard methods and is expected due to the nonlinearity of the underlying biochemical systems. However, depending on the parameter ranges, the adaptive Smolyak algorithm shows in our experiments consistently a significantly better performance than other state of the art methods and thus, is able to deal with the computational forward and Bayesian

inversion analysis even in large-scale applications.

4.3 Model 3

4.3.1 Comparison between the adaptive Smolyak interpolation and a first-order sensitivity approach

A detailed analysis of the sensitivities with respect to the parameters of the model reveals that the sensitivities at the nominal point $\mathbf{p}_0$ of 135 parameters are larger than $1e4$, illustrated in the sensitivity profile of the model in Fig. 4A.

The sensitivity index σ $(n_x = 500,\ n_t = 10)$ for this model equals $2.4860e15$ rendering the forward analysis and Bayesian inference infeasible even for moderate parameter ranges. We therefore restricted the variations of the parameters to the range of ± 0.01 to ensure bounded variations in the states. The Smolyak algorithm fails to converge for larger parameter ranges indicating the ill-conditioning due to modeling issues related to sensitivities of these magnitudes.

As shown in Fig. S18, the estimated error curves of the Smolyak algorithm indicate an asymptotic rate of convergence of 1.5 with respect to the number N of ODE solves needed.

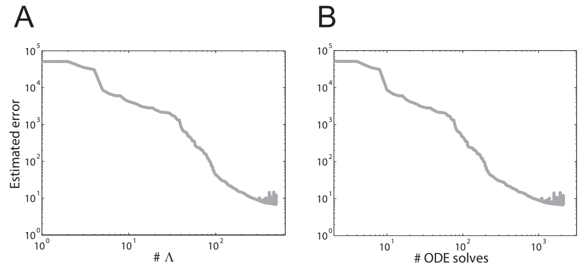

Figure S18. Estimated L^{∞} (absolute) error curves of the adaptive interpolation of state variables $1-500$ with respect to the cardinality of the index set Λ_N based on the sequences CC (left) and with respect to the number of ODE solves needed (right), variations of $\pm 0.01\mathbf{p}_0$, third model.

The comparison of the Smolyak performance and the first-order approximation shows, similar to the previous numerical experiments, a gain in accuracy of up to two orders of magnitude, cf. Fig. S19.

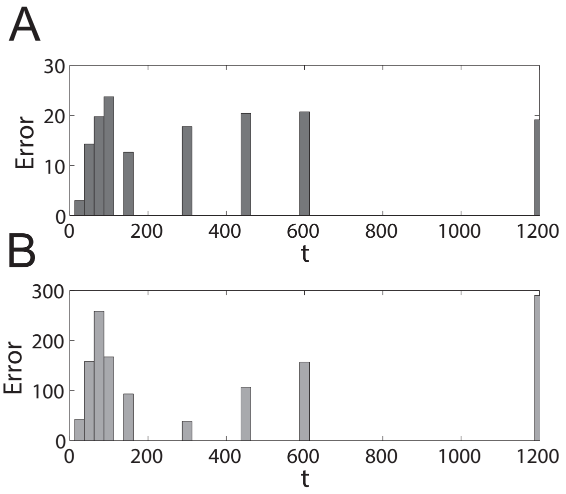

Figure S19. Comparison of the (absolute) errors for the adaptive sparse grid based on CC interpolation nodes (above) and the first-order sensitivity approximation (below), variations of $\pm 0.01\mathbf{p}_0$ (1st realization), third model.

4.3.2 Bayesian inverse problem

We now turn to the inverse problem, i.e. the goal of computation is the conditional expectation of a quantity of interest given noisy observational measurement data. Bearing in mind that the parameter sensitivities are $> 1e5$ for more than half of the parameters, we restrict, as in the previous experiment, the parameter variations to ± 0.01 around their nominal values. The observational noise is assumed to be independently, normally distributed with zero mean and variance $\sigma^2 = 0.1^2$. The proposed adaptive algorithm gives an estimated convergence rate of $1 - 1.5$ in terms of the number N of forward solves, given (artificial) measurement data in the three system variables Akt, Erk, ErbB at time instants $[75.0, 100.0, 150.0]$ (Akt, Erk) and $[50.0, 75.0, 100.0, 150.0]$ (ErbB), see Fig. S20.

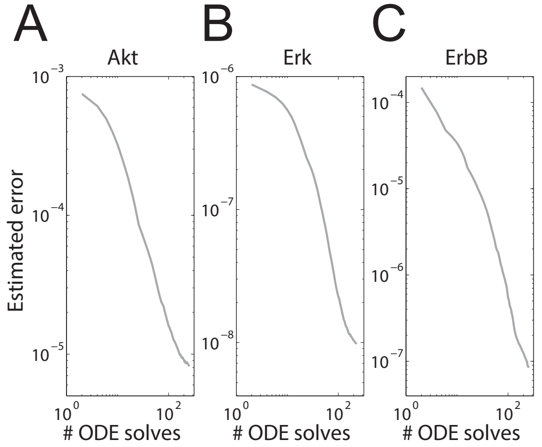

Figure S20. Estimated (absolute) error curves of the normalization constant Z of the three model variables Akt (left), Erk (middle), ErbB (right) with respect to the cardinality of the index set Λ_N (above) based on the sequences CC and with respect to the number of ODE solves needed (below), uniform distribution of the uncertain parameters, variations of $\mathbf{p}_0 \pm 0.01 \cdot \mathbf{p}_0$, third model.

To further explore the parameter space, we propose the following ad-hoc computational remedy, leaving aside the issue of modeling correctness: the idea of the adaptive Smolyak algorithm relies on the efficient detection of dimensions in the parameter space with a large impact on the quantities of interest. The information provided by the already activated indices can be used to enlarge the parameter variations of parameters showing less/ no influence on the quantities of interest, i.e. to enlarge the parameter domain for parameters not activated by the dimension-adaptive Smolyak algorithm. In [18], a reduction of the parameter dimension based on first order sensitivity information is proposed, leading to an overall reduction from 227 to 75 parameters. In contrast to this a priori choice of the active parameters, our approach allows to successively explore and enlarge the parameter space based on the information gained by the adaptive exploration of the parameter space by the Smolyak algorithm.

The number of parameters activated by the algorithm is 112 for variations in the range of $\pm 0.01 \cdot \mathbf{p}_0$. We define a vector $\mathbf{p}_0^1$ by $\mathbf{p}_0^1 \in \mathbb{R}^{227},\ \mathbf{p}_0^1 = 1$, if index i is activated and a second vector $\mathbf{p}_0^2$ by $\mathbf{p}_0^2 \in \mathbb{R}^{227},\ \mathbf{p}_0^2 = 1$, if index i is not activated, indicating the set of parameters with enlarged parameter variations $\pm 0.1\mathbf{p}_0$. The convergence results of this heuristic strategy are summarized in Fig. S21.

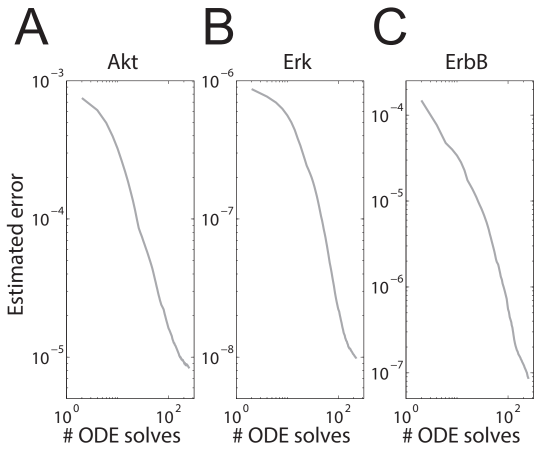

Figure S21. Estimated (absolute) error curves of the normalization constant Z of the three model variables Akt (left), Erk (middle), ErbB (right) with respect to the number of ODE solves needed based on the sequences CC, uniform distribution of the parameters, variations of $\mathbf{p}_0 \pm 0.01 \cdot \mathbf{p}_0^1 \ /\ \pm 0.1 \cdot \mathbf{p}_0^2$, third model.

Repeating the proposed strategy, we define a third vector $\mathbf{p}_0^3$ by $\mathbf{p}_0^3 \in \mathbb{R}^{227},\ \mathbf{p}_0^3 = 1,$ if index i not activated allowing for variations in the range $\pm 1.0\mathbf{p}_0$.

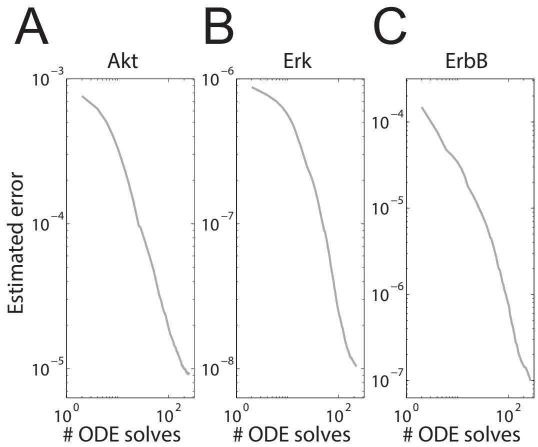

Figure S22. Estimated (absolute) error curves of the normalization constant Z of the three model variables Akt (left), Erk (middle), ErbB (right) with respect to with respect to the number of ODE solves needed based on the sequences CC, uniform distribution of the parameters, variations of $\mathbf{p}_0 \pm 0.01 \cdot \mathbf{p}_0^1 \;/\; \pm 0.1 \cdot \mathbf{p}_0^2 \;/\; \pm 1.0 \cdot \mathbf{p}_0^3$, third model.

As displayed in Fig. S22, we finally end up with a similar convergence behavior as in the case of variations in the range of $\pm 0.01\mathbf{p}_0$ and could moreover significantly enlarge the parameter space. The heuristic strategy proposed above could be a promising approach to exploit the additional information given by the adaptively determined index sets, namely the influence of each parameter on the quantities of interest in the output. The numerical experiments presented clearly indicate the high impact of some parameters on the quantities of interest, but there are also parameters having no or less influence on the response. This nonisotropic behavior can be exploited by the adaptive algorithm, in particular by the proposed heuristic strategy, which allows to enlarge the parameter space. Nevertheless, sensitivities of the magnitude appearing in the sensitivity profile of the underlying model will cause numerical stability problems for any algorithms in system and parameter identification problems. For "reasonable" parameter sensitivities, the proposed dimension-adaptive Smolyak algorithm can construct a parametric surrogate model and allows for computational Bayesian estimation with work vs. accuracy which is dimension-robust (ie., with work which scales linearly with the number of active model parameters) and which can be, asymptotically, superior to that afforded by Monte Carlo algorithms, in all examples which we investigated in the present work. A necessary prerequisite for the success of the presently proposed computational strategy, however, is proper model specification. Model misspecification can severely hamper the performance of any numerical approach. The presently proposed (linear scaling with respect to the model size) computation of "sensitivity profiles" *prior* to Bayesian inversion allows for a fast identification of possible model misspecification and, in any case, alerts the modeler to computational pitfalls due to intrinsic model stiffness and ill-conditioning.

Parameter sensitivities of computed system responses of the magnitude reported in Fig. 4A indicate, in our view, serious issues with numerical stability of forward simulation and calibration, and are to be taken as evidence for model misspecification, which can not (and should not) be addressed by efficient numerical simulation alone.

Assuming the absence of ill-conditioning or of model misspecification, the presently proposed, dimension adaptive interpolation and quadrature algorithms was found to scale and perform reliably in computations for nonlinear models of biochemical reaction pathways with up to several hundred states and parameters. Based on these observations and on recent theoretical results [1] by some of the authors on intrinsic sparsity in

polynomial chaos expansions of the parametric responses, linear scaling and comparable performance are expected for considerably larger models with affine-parametric structure imposed by mass-action kinetics. We add that affine-parametric models are not a prerequisite for the applicability of the presently proposed algorithm. In further experiments, we found similar performance also for Michaelis-Menten kinetics and, based on theoretical results for parametric *partial* differential equation models in [19], analogous (theoretical and computational) results can be expected also for models with nonlinear, holomorphic parametric dependencies.

References

1. Hansen M, Schwab C (2013) Sparse adaptive approximation of high dimensional parametric initial value problems. Vietnam Journal of Mathematics 41: 181–215.

2. Chkifa A, Cohen A, DeVore R, Schwab C (2013) Adaptive algorithms for sparse polynomial approximation of parametric and stochastic elliptic PDEs. M2AN Math Mod and Num Anal 47: 253–280.

3. Chkifa A, Cohen A, Schwab C (2013) High-dimensional adaptive sparse polynomial interpolation and applications to parametric PDEs. Foundations of Computational Mathematics : 1-33.

4. Hansen M, Schillings C, Schwab C (2014) Sparse approximation algorithms for high dimensional parametric initial value problems. Proc of the Fifth International Conference on High Performance Scientific Computing 2012, Hanoi, Vietnam .

5. Cohen A, Chkifa A, Schwab C (2014) Breaking the curse of dimensionality in sparse polynomial approximation of parametric PDEs. Journ Math Pures et Appliquees .

6. Schillings C, Schwab C (2013) Sparse, adaptive smolyak quadratures for Bayesian inverse problems. Inverse Problems 29: 065011.

7. Stuart AM (2010) Inverse problems: A Bayesian perspective. Acta Numerica 19: 451–559.

8. Schwab C, Stuart AM (2012) Sparse deterministic approximation of Bayesian inverse problems. Inverse Problems 28.

9. Schillings C, Schwab C (2014) Sparsity in Bayesian inversion of parametric operator equations. Inverse Problems 30.

10. Gantner RN, Schillings C, Schwab C (2014) Binned multilevel Monte Carlo for Bayesian inverse problems with large data. Domain Decomposition Methods in Science and Engineering XXII .

11. Rizzi M, Theobald U, Querfurth E, Rohrhirsch T, Baltes M, et al. (1996) In vivo investigations of glucose transport in Saccharomyces cerevisiae. Biotechnology and Bioengineering 49: 316–327.

12. Sunnåker M, Schmidt H, Jirstrand M, Cedersund G (2010) Zooming of states and parameters using a lumping approach including back-translation. BMC systems biology 4: 28.

13. Kholodenko BN, Demin OV, Moehren G, Hoek JB (1999) Quantification of short term signaling by the epidermal growth factor receptor. J Biol Chem 274: 30169–30181.

14. Chen WW, Schoeberl B, Jasper PJ, Niepel M, Nielsen UB, et al. (2009) Input-output behavior of ErbB signaling pathways as revealed by a mass action model trained against dynamic data. Molecular Systems Biology 5: 239.

15. Gonnet P, Dimopoulos S, Widmer L, Stelling J (2012) A specialized ODE integrator for the efficient computation of parameter sensitivities. BMC Syst Biol 6: 46.

16. Chib S, Greenberg E (1995) Understanding the Metropolis-Hastings algorithm. The American Statistician 49: 327–335.

17. Gutenkunst RN, Waterfall JJ, Casey FP, Brown KS, Myers CR, et al. (2007) Universally sloppy parameter sensitivities in systems biology models. PLoS Comput Biol 3: 1871–1878.

18. Chen WW, Niepel M, Sorger PK (2010) Classic and contemporary approaches to modeling biochemical reactions. Genes Dev 24: 1861–1875.

19. Hansen M, Schwab C (2013) Analytic regularity and nonlinear approximation of a class of parametric semilinear elliptic PDEs. Mathematische Nachrichten .
